# Daily fluctuation of colonic microbiome in response to nutrient substrates in a pig model

**DOI:** 10.1038/s41522-023-00453-w

**Published:** 2023-11-08

**Authors:** Hongyu Wang, Rongying Xu, Qiuke Li, Yong Su, Weiyun Zhu

**Affiliations:** 1 Laboratory of Gastrointestinal Microbiology, Jiangsu Key Laboratory of Gastrointestinal Nutrition and Animal Health, College of Animal Science and Technology, Nanjing Agricultural University, Nanjing, 210095 China; 2 National Center for International Research on Animal Gut Nutrition, Nanjing Agricultural University, Nanjing, 210095 China

**Keywords:** Microbial ecology, Microbiome

## Abstract

Studies on rodents indicate the daily oscillations of the gut microbiota have biological implications for host. However, the responses of fluctuating gut microbes to the dynamic nutrient substrates are not fully clear. In the study, we found that the feed intake, nutrient substrates, microbiota and metabolites in the colon underwent asynchronous oscillation within a day. Short-chain fatty acids (SCFAs) including acetate, propionate, butyrate and valerate peaked during T24 ~ T27 (Timepoint 24, 12:00 pm, T27, 03:00 am) whereas branched SCFAs isobutyrate and isovalerate peaked during T09 ~ T12. Further extended local similarity analysis (eLSA) revealed that the fluctuation of feed intake dynamically correlated with the colonic carbon substrates which further influenced the oscillation of sugar metabolites and acetate, propionate, butyrate and valerate with a certain time shift. The relative abundance of primary degrader Ruminococcaceae taxa was highly related to the dynamics of the carbon substrates whereas the fluctuations of secondary degraders Lactobacillaceae and Streptococcaceae taxa were highly correlated with the sugar metabolites. Meanwhile, colonic nitrogen substrates were correlated with branched amino acids and the branched SCFAs. Furthermore, we validated the evolution of gut microbes under different carbohydrate and protein combinations by using an in vitro fermentation experiment. The study pictured the dynamics of the micro-ecological environment within a day which highlights the implications of the temporal dimension in studies related to the gut microbiota. Feed intake, more precisely substrate intake, is highly correlated with microbial evolution, which makes it possible to develop chronotherapies targeting the gut microbiota through nutrition intervention.

## Introduction

The mammalian digestive tract harbors trillions of microorganisms whose whole genome is ten times larger than its host^1^. Recently, increasing evidence shows that the microbial consortia undergo spatiotemporal evolution to adapt to the environmental variations in the gut ecosystem^2–6^. Along the temporal dimension, the gut microbiota undergoes seasonal and daily variations in composition and function^6,7^. It is worth noting that the oscillation of the gut microbiota has great importance on the host metabolism, normal physiological rhythm and gut homeostasis^8,9^. However, most of these studies were conducted on rodents. As pigs have significant differences in habits, eating patterns, body size, behavior, life span and gut microbiota compared with rodents. Besides, considering the comparability between pigs and humans in terms of genetic information, anatomic characteristics, eating habits and physiology, observations from the pigs could have a great reference value for human research on gut microbiota^10^.

Intrinsic and extrinsic factors including host genotype, physiology, species, age, gender, environmental factors, feeding pattern and the nutrient composition of feed affect the gut microbiota and its fluctuation^11,12^. Among these factors, feed, more precisely the accessibility and composition of the nutrients, has a significant impact on the composition and function dynamics of the gut microbiota^3,6,7,13,14^. Under the *ad libitum* circumstances, the circadian clock, which synchronizes the behaviors and physiology including food taking and wake-sleep circle, is the most critical factor influencing the gut microbial dynamics^15^. As a result of rhythmic feed-taking behavior, the metabolites in the serum undergo robustly oscillating and are relevant to the gut microbiota fluctuation^9,14,16–18^. The direct effect of dietary interventions functions probably through altering the substrates and metabolome profile in the intestine^19–21^. However, so far, the fluctuation of substrates and metabolites in the colon of growing pigs is unclear. How nutrients shape the gut microbial community and how they drive the metabolic pathways remains unknown.

Of note, light is the primary zeitgeber for the mammalian circadian clock and clear time shifts exist between the zeitgeber and certain physiological phenomena^22,23^. Likely, the effect of nutrient substrate on intestinal microbes may take a specific time to manifest^24,25^. Therefore, it is reasonable to consider the time shift between the substrates and the microbes in their crosstalk. It is of importance for a better understanding of the dynamic microbe-metabolite interactions and providing references for developing a chronotherapy targeting the gut microbiota. However, the dynamics of gut microbes, metabolites profile, their dynamic interactions, as well as the putative time delay remain unclear. Therefore, the present study aimed to discover the fluctuation of colonic substrates, metabolites and the gut microbiota under the free access feeding mode in a pig model and to explore the dynamic microbe-metabolite response to the fluctuation of colonic substrates.

## Results

### Fluctuation of feed intake and colonic substrates within 48 h

**Fig. 1:**
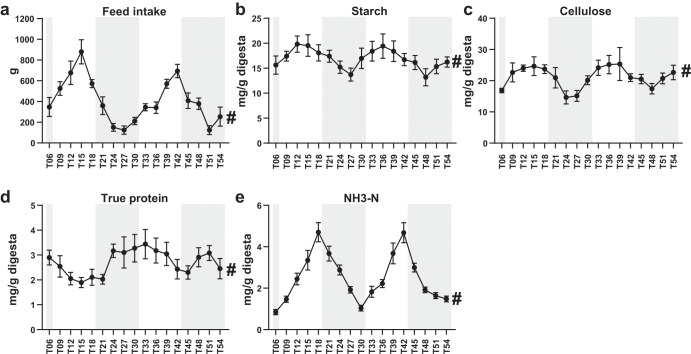
Fluctuation of feed intake and colonic substrates within 48 h. a ~ e Dynamic change of the feed intake during each sampling interval (**a**) and the shifts in concentration of colonic substrate starch (**b**), cellulose (**c**), true protein (**d**) and NH3-N (**e**) at each sampling time point, respectively. # denotes a significant fluctuation within 24 h with a *P*_Adj_ < 0.05. The rhythmicity analysis was finished by non-parametric JTK analysis, *n* = 8. All data were presented as mean ± s.d.

The feed intake of each sampling interval exhibited a rhythmic fluctuation over a course of 48 h (*P*_Adj_ = 3.59 × 10^−15^, Fig. 1a). The feed intake reached a peak at T15 (Timepoint 15, 3:00 pm) and T42 (Timepoint 42, 6:00 pm), whereas reached a trough at T27 and T51. Likewise, the concentrations of colonic starch (*P*_Adj_ = 1.31 × 10^−4^, Fig. 1b) and cellulose (*P*_Adj_ = 7.36 × 10^−6^, Fig. 1c) also underwent robust fluctuation over time with a peak at T12 and T36 or T15 and T39, whereas with a trough at T24 and T48 or T27 and T48. True protein underwent antiphase oscillations with the feed intake with a peak at T06 and T33 and a trough at T15 and T42 (*P*_Adj_ = 0.023, Fig. 1d). The level of NH3-N fluctuated over time with a peak at T18 and T42, whereas with a trough at T06, T30 and T54 (*P*_Adj_ = 2.57 × 10^−32^, Fig. 1e).

### Daily fluctuation of microbial structure in the colon

A total of 3,136,676 sequences summed up to 1 303 584 823 bases were generated with an average sequence of 44,179 ± 1 299 (mean ± standard error) per sample and an average length of 415 bp per sequence (Supplementary Table 2). A phylogenetic tree (Supplementary Fig. 1a) was constructed based on high-quality ASV 16S rRNA gene representative sequences to picture the key taxa in the bacterial community and to exhibit the relative abundance of core ASVs associated with time. The most prevalent phylum in the colon of growing pigs was Firmicutes, followed by Bacteroidetes, Proteobacteria, Actinobacteria and Spirochaetae (Supplementary Fig. 1b). Lactobacillaceae, Ruminococcaceae, Lachnospiraceae and Prevotellaceae represented the dominant families (Supplementary Fig. 1c). The most dominant genera were *Lactobacillus*, *Subdoligranulum*, *Ruminococcaceae_UCG-005*, *Prevotella_9*, *Blautia*, *Alloprevotella, Faecalibacterium* and *Roseburia* (Supplementary Fig. 1d). The rarefaction curve of each group tended to be flat over sampling time (Supplementary Fig. 2a).

**Fig. 2:**
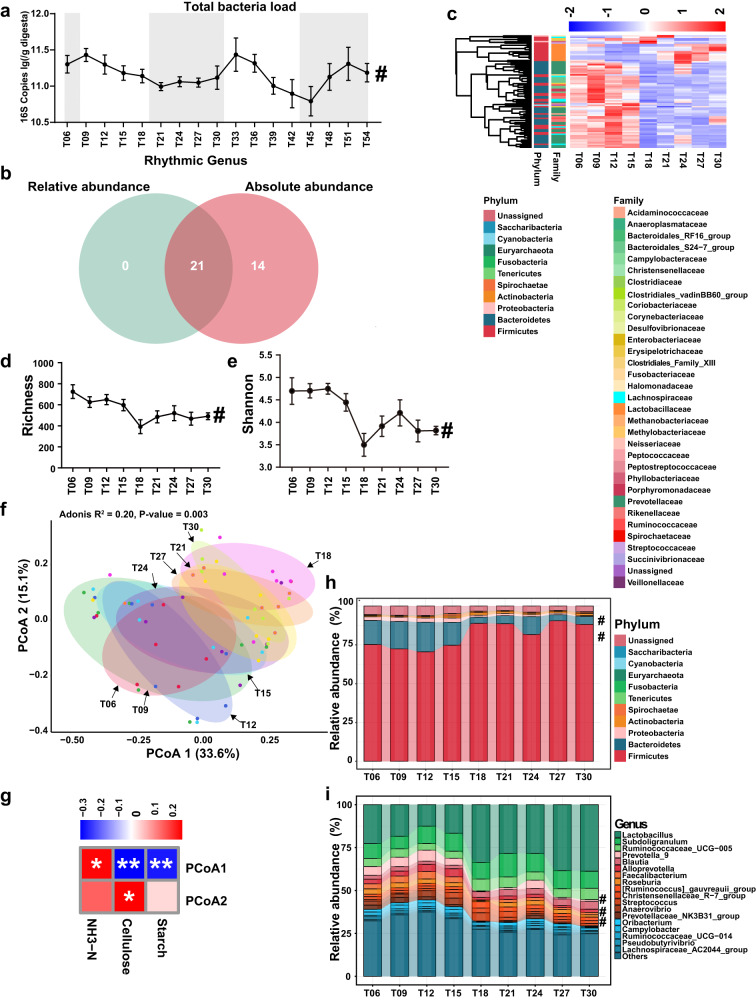
Daily fluctuation of the gut microbiota in the growing pigs. **a** Dynamic change of the total bacterial load at each sampling time point within 48 h. # denotes a significant fluctuation of 24 h with a *P*_Adj_ < 0.05. The rhythmicity analysis was finished by non-parametric JTK analysis with *n* = 8. All data were presented as mean ± s.d. **b** Venn diagram exhibiting the specific number of rhythmic genera identified in the relative abundance and the absolute abundance, respectively. **c** Heatmap depicting the relative abundance of each ASV with rhythmicity. The relative abundance of ASVs was normalized using Z-score methods. Row group annotation information from outside to inside represents its rhythmic fluctuation, corresponding phylum and family, respectively. ASV: amplicon single variant. Dynamic change of alpha diversity induce Richness (**d**) and Shannon (**e**) at different sampling time points. # denotes significant fluctuation with a *P*_Adj_ < 0.05. The rhythmicity analysis was finished by non-parametric JTK analysis with *n* = 8. All data were presented as mean ± s.d. **f** Principal coordinate analysis (PCoA) plot of the bacterial community structure of samples from different time points based on the Bray-Curtis distance. The relative variable importance (R^2^) and significance (*P*) were calculated by PERMANOVA (Adonis) analysis with n = 8. *P* < 0.05 represents a significant difference between different sampling time points. **g** Correlations between colonic substrates and the Bray-Curtis distance of different sampling time points on PCoA1 and PCoA2 based on the Spearman correlation analysis method. * denotes a significant correlation with a *P* < 0.05. Composition and fluctuation of the key gut microbial taxa at the phylum (top 10, **h**) and genus (top20, **i**) level at different time points. # denotes significant rhythmic fluctuation with a *P*_Adj_ < 0.05.

The total bacterial load in colonic digesta underwent significant oscillation over a course of 48 h (*P*_Adj_ = 2.24 × 10^−5^, Fig. 2a) with a peak at the beginning of the light phase and a trough at the beginning of the dark phase. Further, we surprisingly found that all rhythmic genera (Fig. 2b) identified using the relative abundance overlapped with those identified using the absolute abundance. Therefore, the more accessible and convenient relative abundance was mainly discussed in the manuscript.

Non-parameter JTK circle analysis showed that Richness (*P*_Adj_ = 0.046, Fig. 2d) and Shannon index (*P*_Adj_ = 0.011, Fig. 2e) exhibited a significant oscillation with a trough at T18. Consistently, less amount of key microbial genera (mean relative abundance >1.0%) were identified at T18 (Supplementary Fig. 2b). Principal coordinates analysis (PCoA) analysis based on Bray-Curtis dissimilarity differentiated samples from different time points overall (R^2^ = 0.20, *P* = 0.003, Fig. 2f). The result of multiple comparisons indicated that the beta-diversity of T09 and T12 differed significantly from those of T18, T27 and T30 (Supplementary Table 3). Further correlation analysis (Fig. 2g) showed that the first two principal coordinates based on Bray-Curtis distance of samples from different time points were significantly correlated to the colonic substrates starch (PCoA1: *r* = −0.32, *P* = 0.006), cellulose (PCoA1: *r* = −0.36, *P* = 0.002; PCoA2: *r* = 0.25, *P* = 0.037) and the NH3-N (PCoA1: *r* = 0.24, *P* = 0.045).

At the phylum level (Fig. 2h), phyla Firmicutes and Bacteroidetes underwent significant oscillation. The dominant families Prevotellaceae, Streptococcaceae (Supplementary Fig. 2e) and the dominant genera (Fig. 2i, Supplementary Fig. 2f) *Prevotella_1*, *Prevotella_2*, *Prevotella_9*, *Alloprevotella* and *Streptococcus* exhibited significant fluctuation. At the ASV level, 11.22% of ASVs showed significant rhythmic fluctuations (Supplementary Fig. 2c). Heatmap (Fig. 2c and Supplementary Fig. 2g) visualizing the fluctuation of normalized relative abundance of each ASV showed that the relative abundance of all rhythmic ASVs belonging to *Lactobacillus* peaked around T24 during the dark phase, whereas all rhythmic ASVs belonging to Bacteroidetes peaked at T09 ~ T12 during the light phase. We further explored the diurnal difference between the light and dark phases according to the sampling time. At the ASV level, 45 ASVs with a diurnal difference were identified (Supplementary Fig. 2d). Further, the relative abundance of ASVs with diurnal differences belonging to *Lactobacillus* was lower in the light phase than that in the dark phase. In contrast, the relative abundance of differential ASVs belonging to *Prevotella_2*, *Prevotella_7*, *Turicibacter*, *Terrisporobacter* and *Romboutsia* was higher in the light phase than that in the dark phase.

### Daily oscillation of colonic metabolites

**Fig. 3:**
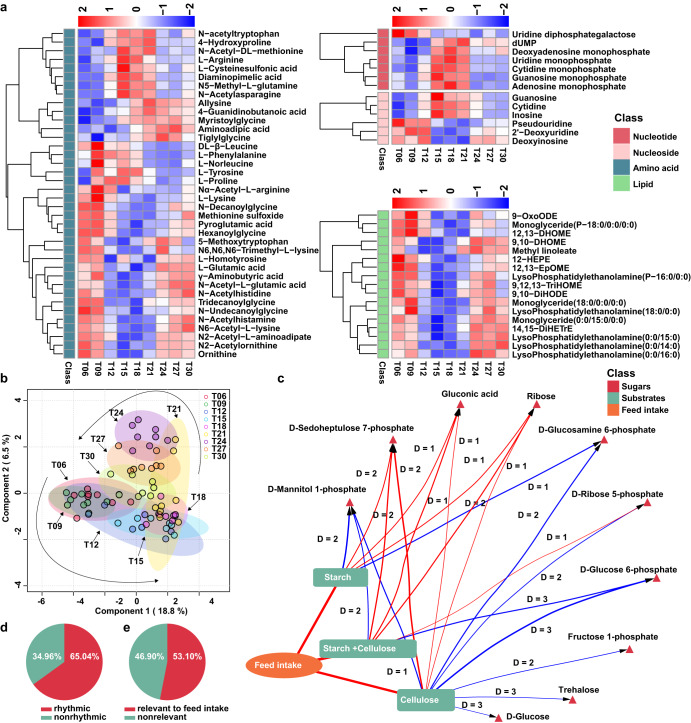
Fluctuation of the metabolites in the colonic digesta of growing pigs. **a** Heatmap depicting the relative abundance of cyclical metabolites of amino acids, lipids, Nucleoside, Nucleotide. The relative abundance was normalized using Z-score methods. Row group annotation information represents the class. **b** Partial least square discriminant analysis (PLS-DA) score plots of the colonic metabolites at different time points within a day (n = 8). **c** Dynamic correlation network between the colonic carbon substrates starch and cellulose, the feed intake and the sugar metabolites based on the extended local similarity analysis (eLSA) method. Each edge represents a pairwise correlation with a local similarity (LS) > 0.5 and a *P* < 0.05. Edges in red color represent positive correlations whereas a blue edge represents a negative correlation. Delay (D) represents time shifts between colonic substrates and sugar metabolites. Pie chart showing the percentage of metabolites with daily fluctuation (**d**) and that relevant to feed intake (**e**).

To clarify the interaction between the gut microbes and the metabolites therein, we further pictured the metabolomic dynamics in the colon of growing pigs. A total of 452 features belonging to amino acids and their derivatives, organic acids, peptides, amines and steroids were identified (Supplementary Fig. 3a). Across the board, the PLS-DA model could distinguish the metabolic profiles of different time points and the score plot exhibited a clear rhythmic pattern (Fig. 3b). Among these metabolites, a total of 294 cyclical features were identified (Supplementary Table 4) which accounted for 65.04% of the total metabolites (Fig. 3d). Of note, metabolites from different substance classes exhibited a tremendous difference in the proportion of metabolites with daily fluctuation varying from 0~100% (Supplementary Fig. 3c). All flavonoids, arachidonic acids, cinnamic acids, quinolines, imidazoles and pyridines exhibited significant oscillations. Whereas more than 70% of peptides, sugars and their derivatives, nucleotides, fatty acids, choline, lipids and vitamins showed rhythmic fluctuation. The relative abundance of most rhythmic amino acids and their derivatives peaked between T15 ~ T18 and T06 ~ T09 (Fig. 3a, Supplementary Fig. 4a). Most peptides, nucleosides, nucleotides and sugars peaked at T15 ~ T18, whereas most lipids reached a nadir during these periods (Fig. 3a, Supplementary Fig. 4a). Accordingly, the abundance of lipids was higher in the dark phase whereas that of peptides and sugars was higher in the light phase (Supplementary Fig. 3d).

Interestingly, 53.10% of all metabolites were closely related to the feed intake, whereas the percentage of rhythmic and nonrhythmic metabolites that correlated to the feed intake was 66.67% and 27.85%, respectively (Fig. 3e, Supplementary Fig. 3f). Notably, we found that the carbon substrates in the colon digesta were closely related to the feed intake (Fig. 3c, Supplementary Fig. 4b). The concentration of the carbon substrates further correlated with the abundance of sugar metabolites with a time shift of 0~3 delay (Fig. 3c). Meanwhile, the amino acids were closely correlated with nitrogen substrates and the feed intakes with a time shift of 0 ~ 3 delay (Supplementary Fig. 4b). Presumably, as a result of the diurnal difference in feed intake, samples taken from the dark phase (T21, T24, T27 and T30) trended to separate from those from the light phase (Fig. 3b). Further, we found that 28.98% of all metabolites exhibited significant diurnal differences (Supplementary Fig. 3e). It was worth noting that 88.55% of these metabolites with diurnal differences underwent rhythmic fluctuation (Supplementary Fig. 3f). In addition, these cyclical metabolites were mainly enriched in pathways of amino acid metabolism, vitamin B6 metabolism, linoleic acid metabolism and starch and sucrose metabolism (Supplementary Fig. 3b).

### Dynamic Microbe-metabolite Interaction in the Colon of Growing Pigs

**Fig. 4:**
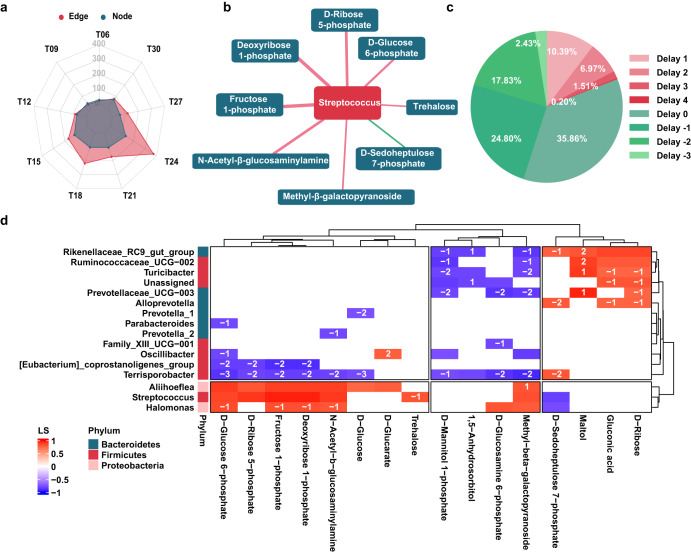
The dynamic microbe-metabolite interaction in the colon of growing pigs within a day. **a** Radar plot showing the number of node and edge in the microbe-metabolite interaction networks at the different time points within a day. **b** Sub-network of microbe-metabolite interaction network showing the detail relationships between *Streptococcus* and sugar metabolites. **c** Pie chart showing the percentage of the correlation pairwise with different time shifts. Pairwise correlation with a negative delay means that the metabolite was ahead of the gut microbe, whereas pairwise correlation with a positive time delay means that the gut microbe was ahead of the metabolite. **d** Dynamic microbe-metabolite interaction network constructed by extended local similarity analysis (eLSA, *n* = 8). Each row represents a genus which were annotated according to the phylum whereas each represents one sugar metabolite. Each cell represents a pairwise correlation with a local similarity (LS) > 0.5 and a *P* < 0.05. The label on the cell represents time delay between a genus and a metabolite. Cells filled in red color represent positive correlations between a genus and a metabolite whereas a blue cell represents a negative correlation. The depth of the color represents the size of LS.

The edge that reflects the microbe-metabolite interaction showed a significant fluctuation (*P*_Adj_ = 0.047) with a peak at T24 during the dark phase (Fig. 4a, Supplementary Fig. 5a). Phyla Firmicutes and Bacteroidetes dominated in the microbe-metabolite interaction networks. Further comparative results for different methods to calculate the correlation between the microbes and metabolites showed that the eLSA method that considered time shift performed better than the Spearman and Pearson method by the numbers of correlation pairwise with statistical significance (Supplementary Fig. 5b). Over 64% of these pairwise with significant correlations had a time shift (Fig. 4c). More specifically, 45.06% pairwise with a negative delay implied that the metabolites influenced the corresponding microbes, whereas the 19.07% pairwise with a positive delay suggested that the microbes may impact the related metabolites. Subnetwork constructed by the eLSA method showed that sugar metabolites had close interactions with microbial genera belonging to Firmicutes, Bacteroidetes and Proteobacteria (Fig. 4d). Especially, *Streptococcus* positively correlated to the sugar metabolites (Fig. 4b, Supplementary Fig. 5c).

### Dynamics of the microbial metabolic function

**Fig. 5:**
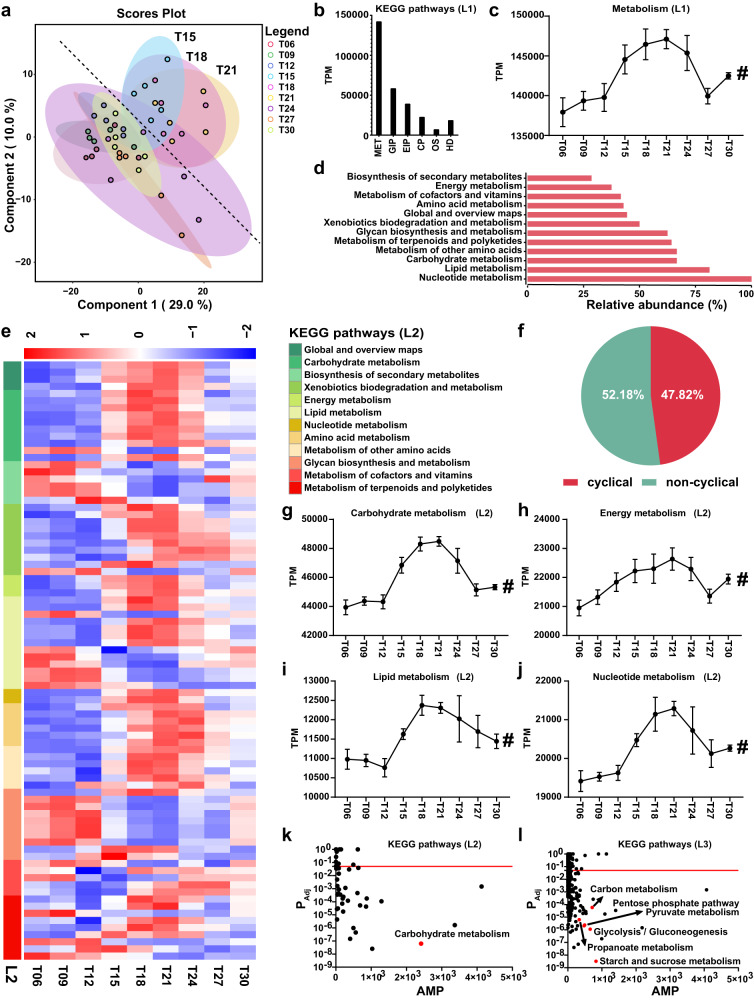
Fluctuation of microbial metabolic functions within a day. **a** Partial least square discriminant analysis (PLS-DA) score plots of the third-level KEGG pathways at different time points in a day. **b** The relative abundances of functional categories at first KEGG pathways. MET = Metabolism, GIP = Genetic information processing, EIP = Environmental information processing; CP = cellular processes; OS = organismal systems and HD = human diseases. TPM = Transcripts per million. **c** Dynamic changes in the relative abundance of metabolism at different time points in a day. # denotes significant daily fluctuation with a *P*_Adj_ < 0.05. The rhythmicity analysis was finished by non-parametric JTK analysis with *n* = 5. TPM = Transcripts per million. **d** Bar chart exhibiting the percentage of the second-level KEGG pathways with rhythmicity of each metabolism category. **e** Heatmap depicting the relative abundance of the third-level KEGG pathways with daily fluctuation. The relative abundance was normalized using Z-score methods. Row group annotation information represents the second-level KEGG pathway category. **f** Pie chart showing the percentage of cyclical KEGG pathways at the third level. Dynamic changes in the relative abundance of carbohydrate metabolism (**g**), energy metabolism (**h**), lipid metabolism (**i**) and nucleotide metabolism (**j**) at the second KEGG pathways at different time points in a day. # denotes significant rhythmicity with a *P*_Adj_ < 0.05. The rhythmicity analysis was finished by non-parametric JTK analysis with *n* = 5. All data were presented as mean ± s.d. TPM = Transcripts per million. Daily fluctuation of the microbial functions at the second-level KEGG pathways (**k**) and third-level KEGG pathways (**l**). The Red dashed line represents a Y-intercept (*P*_Adj_) of 0.05. The rhythmicity analysis was finished by non-parametric JTK analysis with *n* = 5. AMP represents the amplitude (transcripts per million).

Microbial functions were analyzed based on metagenomic sequencing for further revealing the dynamic microbial-metabolite interaction. The PLS-DA model suggested that samples from T15, T18 and T21 tended to cluster together, whereas samples from T24, T27 and T30 generally regressed to those of T06, T09 and T12 (Fig. 5a). About 47.82% of third-level KEGG pathways underwent robust oscillation (Fig. 5f). Metabolism was the most dominant first-level KEGG pathway which peaked at T21 (Fig. 5b, c). The corresponding second-level KEGG pathway had a different cyclical percentage ranging from 27 ~ 100% (Fig. 5d). These metabolism-related pathways exhibited two major rhythmic patterns (peaked at T18 ~ T24 or T09 ~ T12) (Fig. 5e). Most of these pathways peaked at T18 ~ T24, which is consistent with the immediate microbial-metabolite interactions described above. Significantly, the main second-level KEGG pathways related to metabolism include carbohydrate metabolism (Fig. 5g), energy metabolism (Fig. 5h), lipid metabolism (Fig. 5i) and nucleotide metabolism (Fig. 5j) peaked at T21 in the dark phase. Moreover, carbohydrate metabolism-related pathways, including glycolysis/gluconeogenesis, starch and sucrose metabolism, pyruvate metabolism, pentose phosphate pathway, inositol phosphate metabolism and propanoate metabolism exhibited robust oscillation (Figs. 5k, 4l).

### Dynamics of Carbon Flux Distribution and Microbial Succession in the Process of Carbohydrate Metabolism

**Fig. 6:**
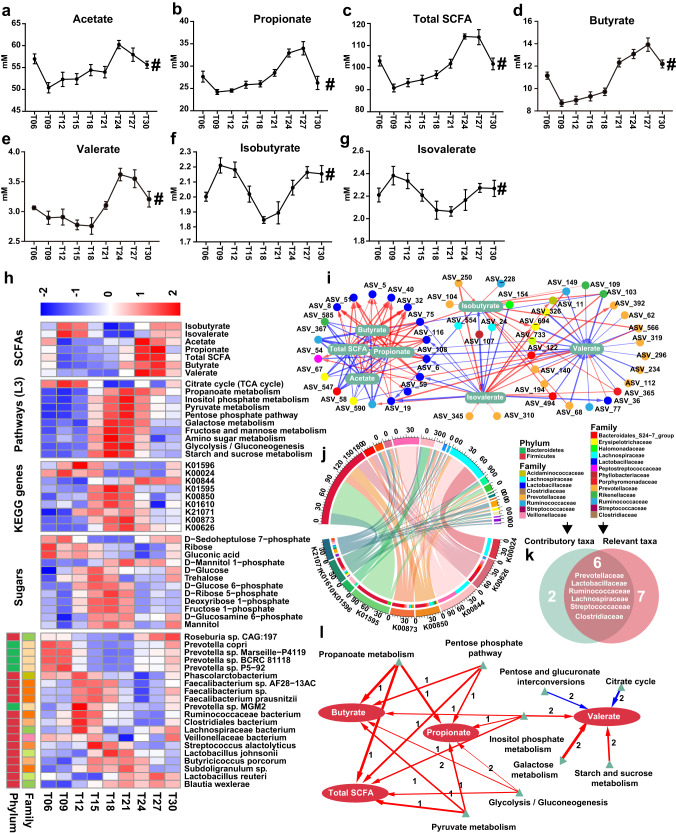
Fluctuations of substances, microbes and pathways in carbohydrate metabolism. Dynamic changes of acetate (**a**), propionate (**b**), total SCFAs (**c**), butyrate (**d**), valerate (**e**), isobutyrate (**f**) and isovalerate (**g**) at different time points. # denotes significant fluctuation with a *P*_Adj_ < 0.05. The rhythmicity analysis was finished by non-parametric JTK analysis with *n* = 8. All data were presented as mean ± s.d. **h** Heatmap depicting the concentration of SCFAs, the abundance of the third-level KEGG pathways, KEGG genes, primary microbial species that contribute to these genes and the sugar metabolites. The abundance was normalized using Z-score methods. **i** Correlation network between the SCFAs and gut microbes at the ASVs level. Each node exhibits an ASV whereas each ellipse represents one of the SCFAs. Each edge represents a correlation with a r > 0.5 and a *P* < 0.05 constructed by eLSA method. All ASVs were colored according to their family. Edges in red color represent positive correlations whereas a blue edge represents a negative correlation. The width of the edge represents the values of the local similarity coefficients. ASV = amplicon single variant. SCFAs = short chain fatty acids. **j** Chord diagram illustrating the main microbial species that contribute to KEGG genes encoding key enzymes involved in carbohydrate metabolism. **k** Venn diagram exhibiting the primary families that tightly correlated to the SCFAs. **l** Correlation between the SCFAs and the carbohydrate metabolism-related pathways. Networks were constructed by eLSA method. Edges in red color represent positive correlations whereas a blue edge represents a negative correlation. The width of the edge represents the values of the local similarity coefficients. The label of the edge represents time shift.

The concentrations of acetate (*P*_Adj_ = 3.21 × 10^−6^, Fig. 6a), propionate (*P*_Adj_ = 2.56 × 10^−9^, Fig. 6b), butyrate (*P*_Adj_ = 1.15 × 10^−14^, Fig. 6d), valerate (*P*_Adj_ = 6.24 × 10^−8^, Fig. 6e), isobutyrate (*P*_Adj_ = 5.67 × 10^−5^, Fig. 6f), isovalerate (*P*_Adj_ = 3.41 × 10^−3^, Fig. 6g) and total SCFAs (*P*_Adj_ = 1.28 × 10^−11^, Fig. 6c) underwent significantly fluctuation. Noteworthy, the concentrations of acetate, propionate, butyrate and valerate mainly from carbohydrate metabolism peaked during T24 ~ T27, whereas isobutyrate and isovalerate mainly from branched amino acids metabolism peaked during T09 ~ T12 (Fig. 6h). eLSA revealed that acetate, propionate, butyrate and the total SCFA were tightly relevant to Lactobacillaceae ASVs and Ruminococcaceae ASVs and valerate was tightly relevant to Prevotellaceae ASVs. In contrast, the branched-chain SCFAs isobutyrate and isovalerate were tightly relevant to Prevotellaceae ASVs and Lachnospiraceae ASVs (Fig. 6i). Further, we found that the SCFAs peaked 1 ~ 2 intervals later than these carbohydrate metabolism-related pathways (Fig. 6l). KEGG genes that encode the key enzymes involved in the biosynthesis of acetate (pyruvate kinase: K00873; phosphofructokinase: K21071 and K00850; hexokinase: K00844), propionate (phosphoenolpyruvate carboxykinase: K01596 and K01610; phosphoenolpyruvate carboxylase: K01595; malate dehydrogenase: K00024) and butyrate (acetyl-CoA C-acetyltransferase: K00626) were mainly contributed by species from Prevotellaceae, Lactobacillaceae, Ruminococcaceae, Lachnospiraceae, Streptococcaceae, Veillonellaceae and Clostridiaceae (Fig. 6j). Interestingly, most of these contributory taxa overlapped with the microbial taxa correlated to the SCFAs (Fig. 6k). The oscillation of Ruminococcaceae taxa including *Faecalibacterium sp*., *Faecalibacterium prausnitzii* and Ruminococcaceae bacterium was highly correlated with the dynamics of the carbon substrates whereas the fluctuation of Lactobacillaceae (*Lactobacillus johnsonii* and *Lactobacillus reuteri*) and Streptococcaceae (*Streptococcus alactolyticus*) were dynamically correlated with the sugar metabolites with a time shift of 0~3 intervals (Supplementary Fig. 6b). Further correlation indicated that the abundance of these KEGG genes was highly correlated with the sugar metabolites (Supplementary Fig. 6a).

### Microbial Succession in Vitro Fermentation Experiment with Different Substrate Combinations

**Fig. 7:**
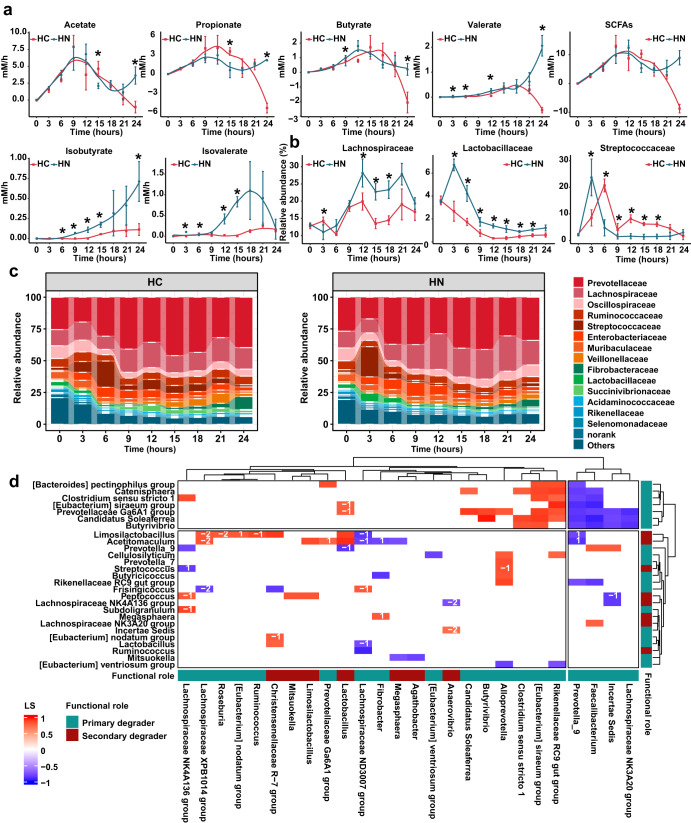
Microbial succession and dynamic fluctuation in short chain fatty acids during in vitro fermentation under different substrate combinations. **a** Production rate of SCFAs acetate, propionate, butyrate, isobutyrate, valerate, isovalerate and total SCFAs. The difference between different groups at each sampling timepoint was identified by a two-tailed *t-test* (*n* = 4). * denotes a significant difference with a *p*-value lower than 0.05. HC = high carbohydrate group; HN = high nitrogen group. All data were presented as mean ± s.d. **b** Dynamic changes in relative abundance of Lachnospiraceae, Lactobacillaceae and Streptococcaceae. The difference between different groups at each sampling timepoint was identified by a two-tailed *t-test* (*n* = 4). * denotes a significant difference with a *p*-value lower than 0.05. HC = high carbohydrate group; HN = high nitrogen group. **c** The microbial composition at each sampling timepoint during in vitro fermentation. HC = high carbohydrate group; HN = high nitrogen group. **d** Correlations among the microbes belonging to different class. Networks were constructed by eLSA method. Each cell represents a pairwise correlation with a local similarity (LS) > 0.5 and a *P* < 0.05. The label on the cell represents time delay of a pairwise. Cells filled in red color represent positive correlations whereas a blue cell represents a negative correlation. The depth of the color represents the size of LS.

In the in vitro fermentation experiment, the bacterial load peaked after a certain time (HC,6 h; HN, 12 h) and reached a steady state thereafter. The bacterial loads of the HN group were higher at 6 (*P* = 0.046), 12 (*P* = 0.015), 21 (*P* = 0.036) and 24 (*P* = 0.007) hours (Supplementary Fig. 7a). Acetate, butyrate, valerate and total SCFAs were higher before 12 h in the HN group whereas the concentrates of propionate and total SCFAs were higher in HC group thereafter (Supplementary Fig. 7b). The concentrates of acetate, propionate, butyrate and valerate peaked during 18 ~ 21 h in the HC group whereas these of HN group remained elevated at 24 h. The concentration of isobutyrate and isovalerate in the HN group were higher than those in HC group (Supplementary Fig. 7b). As expected, the production rates of isobutyrate (6~15 and 24 h) and isovalerate (3, 6, 12 and 15 h) in HN group were higher than those of HC group (Fig. 7a). Correspondingly, the relative abundance of Lactobacillaceae and Streptococcaceae exhibited a rapid increase in the HN group (Fig. 7b, c). Whereas in the HC group, the primary degraders Lachnospiraceae and Ruminococcaceae experienced swift growth, followed by Succinivibrionaceae which peaked at 12 h (Supplementary Fig. 7c). The secondary degrader belonging to Veillonaceae reached their peak at 18:00 (Supplementary Fig. 7c). Further dynamic correlation analysis showed that primary degraders mainly from Lachnospiraceae and Ruminococcaceae peaked 0~2 shift ahead of that of the secondary degrader (Fig. 7d). However, acetate, propionate, butyrate and valerate peaked synchronously with the secondary degraders (Supplementary Fig. 7d).

## Discussion

**Fig. 8:**
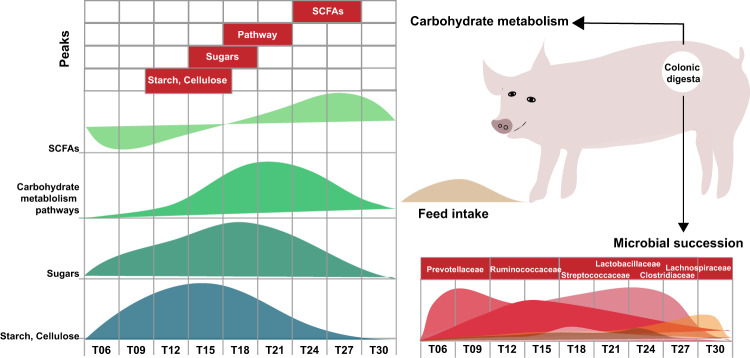
Diagram illustrating carbon flux distribution dynamics and microbial succession in carbohydrate metabolism. In terms of substance metabolism, complex carbohydrate starch and cellulose peaked at T12 ~ T18, monosaccharide (such as glucose) peaked at T15 ~ T21, whereas SCFA (acetate, propionate and butyrate) from bacterial fermentation of carbohydrates peaked at T24 ~ T27. Interestingly, the gut microbial succession with certain nutrients. More specifically, the primary degrader (mainly from Ruminococcaceae and Lachnospiraceae), which play crucial roles in degrading complex carbohydrates to simple monosaccharide, peaked after the complex carbohydrate. Whereas the secondary degrader (mainly from Lactobacillaceae and Streptococcaceae), which play crucial roles in utilizing monosaccharide to produce SCFAs, peaked after the monosaccharide. SCFAs Short chain fatty acids.

The present study aimed to reveal the daily fluctuation of colonic microbe-metabolite interactions in response to the substrates in a pig model. As summarized in Fig. 8, we found that the carbon substrates, sugar metabolites, carbohydrate metabolism-related pathways and the SCFAs production in the colonic digesta of growing pigs underwent asynchronous oscillation within a day. Colonic carbon substrates were dynamically correlated with the course of carbohydrate metabolism and the microbial succession therein. Interestingly, the fluctuation of these microbes had a close correlation with the oscillations of these substrates (metabolites).

Generally, individuals of the rodents were sacrificed for sampling at each time point in time-series studies^7,9,15,26^. Compared with the studies conducted in rodents, the fistula pig model accomplishes consecutive sampling from the same individuals to avoid individual variations in large animals. However, continuous sampling (especially during the night phase) may result in a few tiny differences between the samples and the real condition (such as the feed intake) and small variations in indicators from two consecutive days (such as the total bacterial load). Despite that, these problems are so far inevitable under the present experimental condition.

In the present study, both the nutrients and the microbiota in the colon underwent robust daily fluctuation. Most of the nutrients were highly correlated with the fluctuating feed intakes, thus forming the daily fluctuations in these metabolites. Which could further reprogram the transcriptome in the liver of the host^9^. Specific to microbes in mice, gut microbes belonging to Firmicutes peaked during the activity phase when they were eating and reached a trough during the rest phase. Whereas microbes belonging to Bacteroidetes and Verrucomicrobia underwent antiphase fluctuation^27^. Studies in humans showed that Firmicutes peaked in the light phase (12:00 am), whereas Bacteroidetes peaked in the dark phase (12:00 pm)^28^. In the present study, we found that Firmicutes peaked at T18, which is consistent with the peak time of feed intake. Together, these findings suggest a significant correlation between feed intake and the dynamic fluctuation of gut microbes. Genes that concerning bacterial chemotaxis and flagellar assembly peaked at the end of the rest phase in mice^9^. This may facilitate these microbes permeating to the mucus layer to obtain mucus protein as nutrients.

The daily fluctuation of feed intake under *ad libitum* feeding condition is mainly controlled by the host core circadian clocks^29^. As one of the most important ways of communicating with the external environment and the body, feed intake could reset the peripheral circadian clocks and determine the metabolite profile in the intestine meanwhile^30–32^. Our results showed that the fluctuations of colonic metabolites were tightly correlated with the host feed intakes, suggesting the fluctuating intake of nutrients may further induce the dynamic oscillations of these metabolites. Especially, the fluctuation of the starch and cellulose, which are solely derived from feed, were synchronized with feed intakes. In contrast, the TP concentration was not related to the feed intake. The possible explanation is that most of proteins from feed are absorbed in the proximal small intestine and proteins in the colon mainly derives from endogenous proteins including mucus glycoproteins, sloughed epithelial cells and dead bacteria. A previous study showed that the change in feeding pattern altered the intake of key nutrients and further regulated the milk synthesis rhythm^33^. These polysaccharide carbon substrates in the colon degrade to monosaccharide which is further fermented into SCFAs by the gut microorganisms^34^. Of note, obvious time shifts existed among the carbon substrates, sugars metabolites, carbohydrate metabolic pathways and SCFAs, which reflects the time course of the carbohydrate metabolism. There was a time delay of 0~3 intervals (0~9 h) between the carbon source substrates and sugar metabolites, 0~3 intervals (0~9 h) between the sugar metabolites and carbohydrate pathways (KEGG genes) and 0~2 intervals (0~6 h) between the carbohydrate pathways and SCFAs, respectively. Both in vitro and in vivo studies have demonstrated the time shifts between the substrates, the activities of corresponding metabolic enzymes and microbial metabolites^35,36^. The activities of microbial carbohydrate-active enzymes acetylxylan esterase and arabinofuranosidase, which catalyze the hydrolysis of the polysaccharide, peaked 6 h later after the addition of a specific carbohydrate^35,37^. Whereas the concentration of SCFAs peaked 5 h later after feeding different carbohydrate interventions in the goat’s rumen^36^.

It should be noted that the gut microbiota is the main contributor to the carbohydrate metabolism in the colon. The complex polysaccharide is hydrolyzed by microbial enzymes to generate a series of different metabolites like monosaccharides as mentioned above^34^. These metabolites could further cross-fed other microbes to maintain the homeostasis of intestinal microecology which is of great importance for the host metabolism, health and immunity^38,39^. The microbes play different roles (primary degrader or secondary degrader) in carbohydrate metabolism according to their ecological functions. Interestingly, cumulative evidence indicates that the microbial composition and function fluctuate over a day^7–9,12,27^. Further, we found that the succession of certain microbes in a day was driven by certain substrates as a lot of bacteria have evolved preferences for special substrates^40,41^. For example, Ruminococcaceae taxa *Faecalibacterium*, as a primary degrader that plays a crucial role in the hydrolysis of complex carbohydrates, synchronously reached a peak with the colonic starch and cellulose^42,43^. Whereas the increase of sugar metabolites promoted the proliferation of these secondary degraders *Streptococcus* and *Lactobacillus* which are further fermented to SCFAs^44^. Consistently, the concentrations of SCFAs peaked later than these secondary degraders. Similar to the results in vivo, our in vitro experiment validated that the primary degraders peaked ahead of these secondary degraders with 0~2 shift. However, SCFAs peaked synchronously with the secondary degrader in vitro. The possible explanation was that there may be fundamental differences in the uptake and utilization of nutrient when comparing in vitro and in vivo experiments. Nutrients were more accessible to the microbes in the liquid fermentation cultures. The results of in vitro fermentation experiment also suggest that the microbial metabolic pattern seems to be changing with the substrate. In the presence of ample protein, gut microbes possess the capability to utilize the carbon skeleton derived from protein for the synthesis of SCFAs. This process involves the breakdown of proteins into amino acids via the amino acid metabolic pathway, which are subsequently employed by gut microbes for the synthesis of SCFAs. This might explain the preceded presentation of Lactobacillaceae and Streptococcaceae in the HN group. Also, we established extensive associations between the nutrients and the specific microbes. Therein, we preliminarily validated the response of the overall microbial dynamics to the nutrients in vitro. Of note, followed-up in vivo and in vitro trials to validate these relationships using single-strain bacteria are urgently needed.

The fluctuations of substrates, microbes and microbial metabolites within a day emphasize the necessity to consider the gut micro-ecosystem from a temporal perspective. The dynamic microbe-metabolite interaction network established in the present study suggests that the time shifts between certain gut microbe and metabolites should be taken into consideration when developing a precision regimen targeting the gut microbiota. It also implies that sampling time is a crucial consideration when designing research concerning gut microbiota. *Lactobacillus* ASVs were dominant in the dark phase in this study which implies that the intervention time may affect the effects of specific probiotics. On the other hand, microbial functions related to nutrient metabolism were higher in the dark phase, whereas pathways related to maintenance were higher in the light phase^7^. The abundance of fatty acids and lipids peaked in the dark phase, which was synchronized with their enriched metabolic pathways. Interestingly, the fatty acids in the host serum also peak in the rest phase^45^.

## Methods

### Ethics & Inclusion statement

This project was approved by the Nanjing Agricultural University Animal Care and Use Committee (SYXK2019-0066). All animal care procedures in the experiment were operated according to the Experimental Animal Care and Use Guidelines of China (EACUGC2018-01).

### Animals, experimental design and sampling

**Fig. 9:**
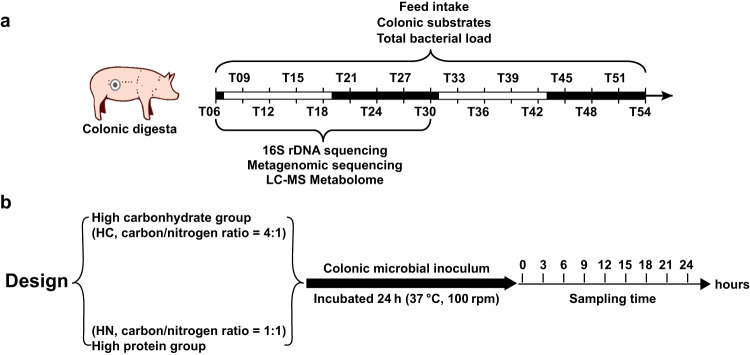
Schematic diagram of the trial design. **a** Schematic diagram of the trial design of the animal experiment. Timepoints within white rectangles represent the light phase, whereas timepoints within black rectangles represent the dark phase. Pigs were fed free access to feed and water throughout the experiment. **b** Schematic diagram of the trial design of in vitro fermentation experiment.

Eight healthy crossbred castrated male pigs (Duroc × Landrace × Large White; average bodyweight ± SE 57.03 ± 1.78 kg, 110 d) were employed in the present study. Pigs were anaesthetized using 3% phenobarbital sodium solution at a dose of 30 mg/kg through the ear vein before the surgery. Each pig was equipped with a T cannula (internal diameter 15 mm, length 82 mm and wings 10 mm) in the proximal colon and raised in an individual pen^46^. All pigs were fed free access to commercial pellets (Supplementary Table 1) for 15 d to adopt the feeding mode under a 12:12 light/dark lightning pattern (light circle: 07:00 am to 07:00 pm). At 06:00 am of the 16^th^ day, colonic digesta were collected at a 3-h interval for consecutive 48 h marked as T06 (06:00 am), T09 (09:00 am), T12 (12:00 am), T15 (03:00 pm), T18 (06:00 pm), T21 (09:00 pm), T24 (12:00 pm), T27 (03:00 am), T30 (06:00 am), T33 (09:00 am), T36 (12:00 am), T39 (03:00 pm), T42 (06:00 pm), T45 (09:00 pm), T48 (12:00 pm), T51 (03:00 am) and T54 (06:00 am), respectively (Fig. 9a). The feed intake of each pig during each sampling interval was also recorded. Unconsumed feed was weighed each sampling timepoint at T03, T06, T09, T12, T15, T18, T21, T24, T30, T33, T36, T39, T42, T45, T48, T51, and T54. Feed intake during each interval was calculated as the difference with previous time point. Digesta samples were snap-frozen in liquid nitrogen for further analysis. Colonic substrates and the total bacterial load were measured for 48 h. As the fluctuation patterns of colonic substrates and the total microbial load within 2 consecutive days were almost consistent. Thus, further 16 S rRNA gene sequencing, metabolic and metagenomic analyses were performed on the samples from the first 24 h.

### In vitro fermentation experiment

To validate the correlation of dynamic fluctuation of microbes with nutrient substrates, a further in vitro fermentation experiment was designed using substrates with different carbon/nitrogen ratio according to the nutritional consumption of the colonic digesta. The high carbon group (HC) consisted of starch (Millipore Sigma), cellulose (Millipore Sigma) and casein (Millipore Sigma) with the mass ratio of 2:2:1 (C/N = 4:1), whereas that of the high nitrogen group (HN) contained the three substrates with the ration of 1:1:2 (C/N = 1:1). Fermentation experiment was carried out using an in vitro batch culture method following Williams, et al.^47^. Colonic microbial inoculum was prepared by diluting the colonic digesta from growing pigs 1:10 with PBS buffer. The inoculum was then filtered by three layers of gauze. One gram of substrate combinations and 10 mL inoculum was added in 90 mL medium. All procedures during the preparation of microbial inoculum and the medium were flushed with CO_2_ to maintain anaerobic conditions. All cultures were incubated at 37 °C in a shaker (100 rpm) for 24 h (Fig. 9b) in anaerobic conditions. Samples were collected every 3 h (0 h, 3 h, 6 h, 9 h, 12 h, 15 h, 18 h, 21 h and 24 h) for the further determination of SCFAs, total bacteria load and 16 S rRNA gene sequencing.

### Measurement of colonic substrates and Short Chain Fatty Acids (SCFAs)

The concentrations of carbon (starch and cellulose) and nitrogen (protein and NH3-N) substrates in the colonic digesta were measured. Starch and cellulose were determined using Solarbio Detection Kit BC0700 (Solaibao, Beijing, China) and Solarbio Detection Kit BC4280 (Solaibao, Beijing, China) respectively referencing the manufacturer’s instructions^48^. The concentration of total protein was determined using Solarbio Detection Kit PC0010 (Solaibao, Beijing, China) through the coomassie brilliant blue G-250 method^49^. The level of NH3-N was measured using the colorimetric method according to our previous method^19^.

SCFAs including acetate, propionate, isobutyrate, butyrate, isovalerate and valerate in the colonic digesta and the fermentation liquids were measured with a gas chromatography method^50^. Agilent 7890A gas chromatograph (Agilent Technologies, Wilmington, DE) equipped with a flame ionization detector was used in the measurement. The production rate of SCFAs in the fermentation liquids were roughly calculated by subtracting the concentrate of the previous timepoint and then divided by sampling interval.

### DNA extraction and the measurement of total bacterial load

Total DNA was extracted from all digesta and the fermentation liquid samples using the cetrimonium bromide method^51^. The total bacterial load (16 S rRNA gene copy) in the colonic digesta was measured using a real-time PCR method^52^. Amplification primer sequences were bacF (5’-CCATTGTAGCACGTGTGTAGCC-3’) and bacR (5’-CGGCAACGAGCGCAACCC-3’). Real-time PCR reactions were performed using a standard SYBR Green PCR kit TSE202 (Tsingke, Beijing, China). Amplification reactions were performed on QuantStudio™ 5 Real-Time PCR Instruments (Applied Biosystems, Foster City, CA) with the standard SYBR cycling amplification conditions. A standard curve method was used to calculate the total bacterial load in the colonic digesta samples. Further, the absolute abundance of each bacterial taxa was obtained by multiplying relative abundance by the total bacterial load.

### 16S rRNA Gene Sequencing and Microbiota Analysis

A universal primer with the unique barcode (forward primer) (5’- CCTAYGGGRBGCASCAG-3’) and reverse primer (5’- GGACTACNNGGGTATCTAAT-3’) was designed to amplify the V3-V4 regions in 16S rRNA gene of the gut microbiota in colonic digesta and the fermentation liquids. Sequencing libraries were estimated using NEB Next^®^Ultra™DNA Library Prep Kit for Illumina (NEB, USA) following the manufacturer’s instruction and the index codes were then added. Library quality was evaluated on the Agilent Bioanalyzer 2100 system and Qubit^@^2.0 Fluorometer (Thermo Scientific). Finally, the library was constructed on an Illumina MiSeq platform and 250 bp/300 bp paired-end reads were generated.

Using VSEARCH (v2.20.1)^53^ paired-end reads of each sample were merged based on the overlaps, primers and barcodes were further trimmed. Reads with an error threshold over 0.01 were then eliminated. Clean reads were further denoised using the UNOISE3 in USEARCH (v10.0.240) after discarding low-abundance noise with a miniquesize of 8 to obtain subsequent amplicon sequence variants (ASVs)^54^. Chimeras were removed by aligned against the Ribosomal Database Project (RDP) (v1.8). Feature ASVs were generated using USEARCH and then were annotated by aligning the taxonomy information against the RDP database using VSEARCH with a confidence of 0.97. Further, non-bacteria and plastids were then removed. Reads count was normalized using the vegan package. Alpha diversity indexes and rarefaction of each group were calculated using USEARCH. A phylogenetic tree based on high-quality sequences was constructed using MUSCLE and iqtree^55,56^ and further visualized using the iTOL online website (https://itol.embl.de/)^57^.

### Microbial functions analysis

A total of 0.5 μg microbial DNA was used for metagenomic shotgun sequencing. Low-quality reads and adaptor contaminants in raw sequence reads were quality trimmed using Trimmomatic^58^. After quality control, reads were then mapped against the human genome (version: hg19) by BWA mem algorithm (parameters: -M -k 32 -t 16, http://bio-bwa.sourceforge.net/bwa.shtml) to remove the host-genome contaminations and low-quality data. For each sample, a set of contigs were generated based on these clean reads using MegaHit (v1.1.3) with parameters of “--min-contig-len 500”^59^. Open reading frames (ORFs) were predicted based on the assembled contigs using Prodigal (v2.6.3)^60^ and all ORFs were generated to a set of unique genes after clustering using CD-HIT (v4.6) with parameters of “-n 9 -c 0.95 -G 0 -M 0 -d 0 -aS 0.9 -r 1”^61^. The longest sequence in each cluster was recognized as the representative sequence in each unique-gene set in each gene. To calculate the abundance of genes within total samples, salmon software (version 0.12.0) was used to obtain the reads number for each gene^62^. This non-redundant gene set was then searched against the Kyoto encyclopedia of genes and genomes (KEGG) databases using BLASTX to identify proteins and annotate their functions. Based on the KO results, the specific function and pathways were obtained using the pathways mapped by the annotated genes based on the KEGG Pathway Database. The gene abundance was calculated finally using the following equations:

1 $$Ab\left( S \right) = Ab\left( U \right) + Ab\left( M \right)$$

2 $$Ab\left( U \right) = \mathop {\sum}\limits_{i = 1}^M {1/l}$$

3 $$Ab\left( M \right) = \mathop {\sum}\limits_{i = 1}^M {\left( {Co \ast 1} \right)/l}$$

4 $$Co = \frac{{Ab\left( U \right)}}{{\mathop {\sum}\nolimits_{i = 1}^N {Ab\left( {U_i} \right)} }}$$

*Ab*(*S*), gene abundance; *Ab*(*U*), single-mapping reads abundance; *Ab*(*M*), multi-mapping reads abundance; *l*, length of gene sequence^63^. The abundance values in metagenomes were normalized by transcripts per kilobase per million mapped reads (TPM).

### Metabolome analysis

Metabolome analysis was finished based on the liquid chromatography-mass spectrometry method. The liquid chromatography-mass spectrometry platform was based on Ultimate 3000LC (Thermo, Q Exactive) equipped with a Hypersil GOLD™ C18 Column (100 mm × 2.1 mm, 1.9 µm). The detailed parameters were described elsewhere^19^. Spectrum information was parsed using Compound Discoverer software 3.0 (Thermo Scientific). Downstream data were analyzed using MetaboAnalyst 5.0 (https://www.metaboanalyst.ca/)^64^. Auto-scaling normalization and logarithmic transformation were executed after QC adjustment. The partial least squares-based discriminant analysis (PLS-DA) method was used to picture the overall variations among different time points. KEGG pathway enrichment analysis was processed to discover the enriched pathways of features with daily fluctuation.

### Data Analysis and Statistics

An R package JTK_circle analysis was used to detect the daily fluctuation with a period of 24 h and an interval of 3 h^7,65^. Using this algorithm, permutation-based *P* (*P*_Adj_) and amplitude (AMP) were given. Features with *P*_Adj_ < 0.05 were considered to have daily fluctuation (95% CI). Only microbial taxa with a relative abundance over 0.01% and presented in more than 20% of samples were retained for further analysis. The microbial-metabolite network for each sampling time point was constructed using Spearman’s correlation method. Correlations with a correlation coefficient (R) higher than 0.5 and a p-value lower than 0.05 were retained. Extended local similarity analysis (eLSA) was used to calculate the correlations among feed intake, cyclical microbial taxa, cyclical metabolites, SCFAs, KEGG genes along with their contributory taxa and KEGG pathways (95% CI)^66^. The local similarity coefficient (LS) was used to measure the correlation level pairwise. A delay value (D) was calculated to represent the time shift between a pairwise. Correlations with a LS higher than 0.5 and a p-value lower than 0.05 will be retained for further analysis. Cytoscape (v3.7.2) and gephi (v0.9.4) were used to visualize the microbe-metabolite interaction networks. STAMP (v 2.1.330) was used to explore the diurnal differences in the composition and function of the gut microbiota and the metabolites between the light phase (T09, T12, T15 and T18) and the dark phase (T06, T21, T24, T27 and T30)^67^. The difference between different groups at each sampling timepoint was identified by a two-tailed *t*-test using SPSS (IBM SPSS 21.0, SPSS Inc.). *P* values less than 0.05 (*P* < 0.05) indicated significant differences (95% CI).

### Reporting summary

Further information on research design is available in the Nature Research Reporting Summary linked to this article.

### Supplementary information

Supplementary information

Reporting summary

## Data Availability

The datasets generated and/or analyzed during the current study are available in the NCBI Sequence Read Archive database repository, https://dataview.ncbi.nlm.nih.gov/object/PRJNA824879 and https://dataview.ncbi.nlm.nih.gov/object/PRJNA843783.

## Code availability

Codes utilized for the analysis and visualization of the study data were included in the supplementary information of this manuscript.
