# Supplementary material for: Daily fluctuation of colonic microbiome in response to nutrient substrates in a pig model
Source: NPJ Biofilms Microbiomes. 2023 Nov 8;9:85. doi: 10.1038/s41522-023-00453-w (PMC10632506; doi:10.1038/s41522-023-00453-w)
Supplement: Supplementary file 1 — Supplementary information [file 41522_2023_453_MOESM1_ESM.pdf]

Supplementary information

Supplementary Table 1 Ingredients composition and calculated nutritional level of the experiment diet (as-fed basis)

Ingredient	Percentage (%)	Calculated nutritional compositions (%)	
Corn	70.0	Digestive energy（MJ/kg）	14.60
Soybean meal	18.0	Crude protein	16.00
Wheat bran	6.50	Lysine	1.23
Soybean oil	1.90	Methionine+Cystine	0.70
Lysine	0.69	Threonine	0.79
Methionine	0.24	Tryptophan	0.22
Threonine	0.30		
Tryptophan	0.07		
Calcium hydrogen phosphate	0.45		
Stone powder	0.50		
Salt	0.30		
Multivitamins[1]	0.03		
Minerals[2]	0.20		
Choline chloride (50%)	0.12		
Zeolite powder	0.60		
Antioxidant	0.1		
Total	100.0		

[1] The minerals supply per kg diet as follows: Fe 165 mg, Zn 165 mg, Cu 16.5 mg, Mn 30 mg, Co 0.15 mg, I 0.25 mg, Se 0.25 mg.

[2] The multivitamins supply per kg diet as follows: VA 11 000 IU, VD3 1 000 IU, VE 16 IU, VK1 1mg, VB1 0.6 mg, VB2 0.6 mg, d-pantothenic acid 6 mg, nicotinic acid 10 mg, VB12 0.03 mg, folic acid 0.8 mg, VB6 1.5 mg.

Supplementary Table 2 16S rDNA sequencing data statistics

Sample	Sequences	Bases (bp)	Average Length (bp)
T12_1	56216	23272762	413.99
T12_2	51924	21442364	412.96
T12_3	33194	13723904	413.45
T12_4	49292	20455127	414.98
T12_5	42350	17623721	416.14
T12_6	50712	21064714	415.38
T12_7	32536	13584379	417.52
T12_8	56112	23159366	412.73
T15_1	40442	16895191	417.76
T15_2	52788	21947454	415.77
T15_3	31038	12778680	411.71
T15_4	45542	18935721	415.79
T15_5	34311	14199565	413.85
T15_6	32898	13730704	417.37
T15_7	43326	18046926	416.54
T15_8	59704	24769857	414.88
T18_1	50271	20571309	409.21
T18_2	45071	18904656	419.44
T18_4	48453	20281457	418.58
T18_5	57804	23866375	412.88
T18_6	58453	24457950	418.42
T18_7	44719	18499951	413.69
T18_8	59709	25114141	420.61
T21_1	41173	16843597	409.09
T21_2	37835	15765985	416.7
T21_3	46344	19296769	416.38
T21_4	31586	13125668	415.55
T21_5	42596	17567074	412.41
T21_6	36520	15192922	416.02
T21_7	50881	21096941	414.63
T21_8	45853	19036609	415.17
T24_1	37550	15390522	409.87
T24_2	44779	18396463	410.83
T24_3	50058	20868204	416.88
T24_4	41136	17107602	415.88
T24_5	42462	17606535	414.64
T24_6	58122	24649930	424.11
T24_7	55333	23116358	417.77
T24_8	33479	13885917	414.76

T27_1	53795	22205496	412.78
T27_2	35237	14601139	414.37
T27_3	32236	13444076	417.05
T27_4	53349	22161610	415.41
T27_5	49776	20517407	412.19
T27_6	56971	24029429	421.78
T27_7	56050	23356207	416.7
T27_8	32303	13541044	419.19
T30_1	54635	22583492	413.35
T30_2	38071	15785162	414.62
T30_3	30636	12794414	417.63
T30_4	44042	18383441	417.41
T30_5	51690	21542644	416.77
T30_6	44432	18560115	417.72
T30_7	39457	16443511	416.75
T30_8	54311	22688997	417.76
T06_1	46419	19221808	414.09
T06_2	34358	14272713	415.41
T06_3	33270	13842207	416.06
T06_4	49467	20589601	416.23
T06_5	34022	14093994	414.26
T06_6	30975	12816787	413.78
T06_7	43899	18134018	413.08
T06_8	57876	24404492	421.67
T09_1	51670	21536233	416.8
T09_2	53934	22390376	415.14
T09_3	43186	17839928	413.1
T09_4	59488	24608507	413.67
T09_5	11876	4922795	414.52
T09_6	13890	5772307	415.57
T09_7	16852	7013280	416.17
T09_8	55931	23214223	415.05
Total	3136676	1.3E+09	--
Average	44178.54	18360350	415.5
SE[1]	1290.05	538810.8	0.32

[1] SE represents standard error.

Supplementary Table 3 Multiple comparisons between samples from different time points

Pairs	F Model	P value
T06 vs T18	3.551	0.019
T06 vs T30	2.9	0.025
T09 vs T18	3.47	0.006
T09 vs T27	2.426	0.032
T09 vs T30	3.191	0.017
T12 vs T18	3.993	0.004
T12 vs T27	2.763	0.015
T12 vs T30	3.725	0.003
T15 vs T18	2.613	0.02
T15 vs T30	2.676	0.009
T18 vs T24	2.539	0.022
T18 vs T27	1.918	0.066
T18 vs T30	1.807	0.071
T06 vs T27	2.135	0.078
T09 vs T21	2.026	0.082
T12 vs T21	1.804	0.087
T15 vs T27	1.834	0.091
T06 vs T21	1.719	0.098
T24 vs T30	1.608	0.119
T18 vs T21	1.624	0.144
T21 vs T30	1.201	0.252
T12 vs T24	1.099	0.343
T09 vs T24	0.999	0.379
T15 vs T21	0.964	0.395
T15 vs T24	0.924	0.472
T06 vs T24	0.846	0.545
T09 vs T15	0.793	0.593
T06 vs T15	0.747	0.671
T24 vs T27	0.679	0.674
T21 vs T24	0.574	0.79
T21 vs T27	0.484	0.824
T27 vs T30	0.503	0.897
T06 vs T12	0.452	0.955
T09 vs T12	0.339	0.96
T12 vs T15	0.312	0.963
T06 vs T09	0.266	0.988

Supplementary Table 4 Cyclical metabolites identified by JTK_circle algorithm

NO.	Feature	Class	P_{Adj}[1]
1	Proline betaine	alkaloids	9.90E-08
2	Xanthine	alkaloids	2.61E-04
3	6-Hydroxynicotinic acid	alkaloids	4.68E-04
4	Hypoxanthine	alkaloids	5.11E-04
5	DL-pipecolic acid	alkaloids	6.71E-03
6	Spermidine	amine	2.15E-06
7	N-Acetylputrescine	amine	4.00E-05
8	Tetradecylamine	amine	4.00E-05
9	Porphobilinogen	amine	3.74E-04
10	Sphingosine	amine	3.97E-03
11	L-Prolinamide	amine	1.92E-02
12	N,N-Dimethylaniline	amine	2.68E-02
13	N,N-Dimethylsphingosine	amine	2.95E-02
14	1-Methylhistamine	amine	3.10E-02
15	Sphinganine	amine	3.14E-02
16	N8-Acetylspermidine	amine	3.46E-02
17	Alpha-Linolenoyl ethanolamide	amine	3.92E-02
18	Diaminopimelic acid	amino acids and their derivatives	4.01E-07
19	N-Acetylhistamine	amino acids and their derivatives	4.54E-07
20	N5-Methyl-L-glutamine	amino acids and their derivatives	6.96E-07
21	Pyroglutamic acid	amino acids and their derivatives	8.35E-07
22	Methionine sulfoxide	amino acids and their derivatives	2.03E-06
23	N-Acetyl-L-glutamic acid	amino acids and their derivatives	2.03E-06
24	Allysine	amino acids and their derivatives	1.66E-05
25	Hexanoylglycine	amino acids and their derivatives	2.80E-05
26	N6-Acetyl-L-lysine	amino acids and their derivatives	4.00E-05
27	N2-Acetyl-L-aminoadipate	amino acids and their derivatives	4.65E-05
28	L-Lysine	amino acids and their derivatives	6.27E-05
29	N-Acetylasparagine	amino acids and their derivatives	8.41E-05
30	L-Arginine	amino acids and their derivatives	9.27E-05
31	N2-Acetylornithine	amino acids and their derivatives	1.89E-04
32	5-Methoxytryptophan	amino acids and their derivatives	2.86E-04
33	N-acetyltryptophan	amino acids and their derivatives	3.58E-04
34	N-Acetylhistidine	amino acids and their derivatives	5.34E-04
35	L-Cysteinesulfonic acid	amino acids and their derivatives	6.92E-04
36	4-Hydroxyproline	amino acids and their derivatives	1.15E-03
37	L-Phenylalanine	amino acids and their derivatives	1.30E-03
38	N-Decanoylglycine	amino acids and their derivatives	2.39E-03
39	Aminoadipic acid	amino acids and their derivatives	3.41E-03

40	4-Guanidinobutanoic acid	amino acids and their derivatives	5.18E-03
41	Tiglylglycine	amino acids and their derivatives	5.18E-03
42	Tridecanoylglycine	amino acids and their derivatives	7.77E-03
43	DL-beta-Leucine	amino acids and their derivatives	8.35E-03
44	Ornithine	amino acids and their derivatives	9.64E-03
45	N-Undecanoylglycine	amino acids and their derivatives	1.11E-02
46	Myristoylglycine	amino acids and their derivatives	1.15E-02
47	L-Glutamic acid	amino acids and their derivatives	1.32E-02
48	L-Homotyrosine	amino acids and their derivatives	1.32E-02
49	N6,N6,N6-Trimethyl-L-lysine	amino acids and their derivatives	1.52E-02
50	L-Tyrosine	amino acids and their derivatives	2.20E-02
51	L-Norleucine	amino acids and their derivatives	2.39E-02
52	N-Acetyl-DL-methionine	amino acids and their derivatives	3.80E-02
53	gamma-Aminobutyric acid	amino acids and their derivatives	3.80E-02
54	L-Proline	amino acids and their derivatives	4.44E-02
55	Nalpha--Acetyl-L-arginine	amino acids and their derivatives	4.44E-02
56	Thymine	bases	6.96E-07
57	Uracil	bases	9.73E-06
58	Cytosine	bases	1.43E-04
59	Gentisic acid	benzoic acid and its derivatives	8.01E-05
60	Salicylic acid	benzoic acid and its derivatives	9.27E-05
61	Benzoic acid	benzoic acid and its derivatives	3.58E-04
62	Butylparaben	benzoic acid and its derivatives	1.92E-02
63	Hippuric acid	benzoic acid and its derivatives	4.73E-02
64	Biliverdin	bilirubin	6.27E-05
65	Bilirubin	bilirubin	8.58E-04
66	cis-5-Tetradecenoylcarnitine	carnitine	9.74E-04
67	3-Hydroxytetradecanoyl carnitine	carnitine	2.92E-03
68	3-Hydroxyhexadecanoylcarnitine	carnitine	3.54E-03
69	3-Hydroxyhexadecadienoylcarnitine	carnitine	3.35E-02
70	Glycerophosphocholine	choline	3.54E-03
71	Acetylcholine	choline	8.97E-03
72	Choline	choline	3.25E-02
73	Caffeic acid	cinnamic acid class	4.68E-04
74	hydrocinnamic acid	cinnamic acid class	6.92E-04
75	Sinapic acid	cinnamic acid class	1.81E-03
76	2-Hydroxycinnamic acid	cinnamic acid class	1.03E-02
77	Cinnamic acid	cinnamic acid class	1.47E-02
78	Prostaglandin B1	eicosanoid	1.91E-06
79	15-Keto-13,14-dihydroprostaglandin A2	eicosanoid	3.21E-06
80	Prostaglandin E3	eicosanoid	3.61E-05
81	8-iso-15-keto-PGF2a	eicosanoid	4.42E-05

82	Prostaglandin A2	eicosanoid	1.89E-04
83	Prostaglandin F1a	eicosanoid	6.24E-03
84	alpha-Linolenic acid	fatty acids	5.13E-07
85	Hexadecanedioic acid	fatty acids	7.83E-06
86	Myristoleic acid	fatty acids	4.42E-05
87	Stearic acid	fatty acids	1.12E-04
88	Oleic Acid	fatty acids	1.43E-04
89	Palmitelaidic acid	fatty acids	1.89E-04
90	Docosahexaenoic acid	fatty acids	2.61E-04
91	Linoelaidic acid	fatty acids	4.89E-04
92	Arachidonic Acid	fatty acids	8.58E-04
93	Lauric acid	fatty acids	1.15E-03
94	15-Methylpalmitate	fatty acids	1.23E-03
95	Eicosapentaenoic acid	fatty acids	1.88E-03
96	Adrenic Acid	fatty acids	2.13E-03
97	Linoleic acid	fatty acids	2.21E-03
98	Palmitic acid	fatty acids	3.97E-03
99	Tetracosahexaenoic acid	fatty acids	5.18E-03
100	Dihomo-gamma-linolenic acid	fatty acids	4.44E-02
101	(R)-Equol	flavonoids	4.54E-07
102	Phloretin	flavonoids	1.06E-06
103	Genistein	flavonoids	1.34E-05
104	Luteolin	flavonoids	1.49E-05
105	Daidzein	flavonoids	4.65E-05
106	Diosmetin	flavonoids	7.26E-05
107	Isokaempferide	flavonoids	2.07E-04
108	Glycitein	flavonoids	6.63E-04
109	Kaempferol	flavonoids	1.02E-03
110	Genistin	flavonoids	4.29E-03
111	Naringenin	flavonoids	6.24E-03
112	Chrysin	flavonoids	1.15E-02
113	Hesperetin	flavonoids	2.20E-02
114	Daidzin	flavonoids	2.43E-02
115	Urocanic acid	imidazoles	1.96E-03
116	N-Methylhydantoin	imidazoles	1.19E-02
117	5-Hydroxyindoleacetic acid	indoles	4.20E-05
118	Indole-3-carboxylic acid	indoles	6.27E-05
119	Indole-3-methyl acetate	indoles	4.28E-04
120	5-Methoxyindole	indoles	5.57E-04
121	5-Hydroxyindole	indoles	4.29E-03
122	Indoxyl	indoles	5.18E-03
123	Indoleacetic acid	indoles	9.98E-03

124	Phosphoric acid	Inorganic acid	6.91E-05
125	9,10-DHOME	lipid	1.06E-06
126	14,15-DiHETrE	lipid	2.55E-06
127	Methyl linoleate	lipid	2.70E-06
128	Monoglyceride(0:0/15:0/0:0)	lipid	9.73E-06
129	12-HEPE	lipid	1.57E-05
130	LysoPhosphatidylethanolamine(P-16:0/0:0)	lipid	1.94E-05
131	Monoglyceride(P-18:0/0:0/0:0)	lipid	8.01E-05
132	Monoglyceride(18:0/0:0/0:0)	lipid	1.30E-04
133	12,13-DHOME	lipid	6.92E-04
134	LysoPhosphatidylethanolamine(0:0/14:0)	lipid	1.25E-03
135	LysoPhosphatidylethanolamine(0:0/16:0)	lipid	1.48E-03
136	9-OxoODE	lipid	2.30E-03
137	9,12,13-TriHOME	lipid	3.41E-03
138	9,10-DiHODE	lipid	3.82E-03
139	LysoPhosphatidylethanolamine(18:0/0:0)	lipid	4.62E-03
140	12,13-EpOME	lipid	5.58E-03
141	LysoPhosphatidylethanolamine(0:0/15:0)	lipid	4.73E-02
142	Cytidine	nucleoside	4.50E-06
143	Guanosine	nucleoside	6.92E-04
144	Inosine	nucleoside	1.81E-03
145	2'-Deoxyuridine	nucleoside	6.47E-03
146	Deoxyinosine	nucleoside	1.02E-02
147	Pseudouridine	nucleoside	2.76E-02
148	Uridine monophosphate	nucleotide	2.03E-06
149	Cytidine monophosphate	nucleotide	3.21E-06
150	Guanosine monophosphate	nucleotide	1.02E-04
151	dUMP	nucleotide	1.64E-04
152	Adenosine monophosphate	nucleotide	3.27E-04
153	Uridine diphosphategalactose	nucleotide	9.30E-03
154	Deoxyadenosine monophosphate	nucleotide	1.52E-02
155	Ricinoleic acid	organic acids	2.27E-06
156	3-Hydroxyglutaric acid	organic acids	7.41E-06
157	Adipic acid	organic acids	9.73E-06
158	ferulic acid	organic acids	1.18E-04
159	2-Hydroxy myristic Acid	organic acids	2.07E-04
160	Chaulmoogric acid	organic acids	2.86E-04
161	10-Nitrolinoleic acid	organic acids	3.42E-04
162	3-Hydroxymethylglutaric acid	organic acids	4.89E-04
163	Aminocaproic acid	organic acids	7.88E-04
164	Methyl acetoacetate	organic acids	8.22E-04
165	16-Hydroxy hexadecanoic acid	organic acids	8.58E-04

166	3-Oxohexadecanoic acid	organic acids	1.74E-03
167	Oxoadipic acid	organic acids	1.74E-03
168	Mevalonic acid	organic acids	2.13E-03
169	Undecanedioic acid	organic acids	3.28E-03
170	3-Hydroxysebacic acid	organic acids	4.13E-03
171	3-Hydroxysuberic acid	organic acids	4.45E-03
172	Tetradecanedioic acid	organic acids	6.96E-03
173	2-Isopropylmalic acid	organic acids	7.49E-03
174	Dodecanedioic acid	organic acids	8.35E-03
175	Oxoglutaric acid	organic acids	8.65E-03
176	3-hydroxypentadecanoic acid	organic acids	1.11E-02
177	Suberic acid	organic acids	1.32E-02
178	3-Hydroxy dodecanedioic acid	organic acids	1.42E-02
179	Undecylenic acid	organic acids	1.57E-02
180	3,3-Dimethylglutaric acid	organic acids	1.57E-02
181	12-Hydroxystearic acid	organic acids	2.16E-02
182	Citric acid	organic acids	3.05E-02
183	3-Hydroxytetradecanedioic acid	organic acids	3.35E-02
184	Quinic acid	organic acids	3.80E-02
185	Maleamic acid	organic acids	4.44E-02
186	Methyl Arachidonate	other	1.27E-06
187	N-Acetylcadaverine	other	1.34E-05
188	Phenylpyruvic acid	other	9.27E-05
189	Vanilpyruvic acid	other	4.09E-04
190	2-Oxoarginine	other	1.36E-03
191	N-Acetylmuramic acid	other	1.48E-03
192	4-Hydroxybenzaldehyde	other	2.21E-03
193	N-Desmethyltramadol	other	2.75E-03
194	Dihydroferulic acid	other	2.92E-03
195	Picolinic acid	other	9.98E-03
196	acetophenone	other	1.19E-02
197	Phenylacetaldehyde	other	1.32E-02
198	L-3-Phenyllactic acid	other	2.13E-02
199	Vanillactic acid	other	2.68E-02
200	2-Oxazolidinone	other	3.35E-02
201	N-Acetylneuraminic acid	other	4.87E-02
202	Serylproline	peptides	7.12E-08
203	Asparaginylisoleucine	peptides	2.28E-07
204	Valyl-Valine	peptides	3.54E-07
205	Prolylleucine	peptides	9.41E-07
206	Asparaginyl-Valine	peptides	2.27E-06
207	Valylleucine	peptides	2.55E-06

208	Alanylleucine	peptides	2.70E-06
209	Serinyl-Leucine	peptides	2.70E-06
210	Prolyl-Tryptophan	peptides	6.64E-06
211	Gamma-Glutamylglutamine	peptides	7.41E-06
212	Alanyl-Proline	peptides	8.26E-06
213	Alanyl-Tyrosine	peptides	8.26E-06
214	Arginylleucine	peptides	1.08E-05
215	Alanylvaline	peptides	1.14E-05
216	Leucylleucine	peptides	2.39E-05
217	Leucylphenylalanine	peptides	3.80E-05
218	gamma-Glutamylglycine	peptides	6.58E-05
219	Valyl-Proline	peptides	6.58E-05
220	Glycyl-Phenylalanine	peptides	8.01E-05
221	Glycylproline	peptides	1.18E-04
222	Aspartylphenylalanine	peptides	1.49E-04
223	Leucylproline	peptides	2.38E-04
224	Leucyl-Tryptophan	peptides	2.49E-04
225	Alanyl-Tryptophan	peptides	2.61E-04
226	Alanyl-Glutamine	peptides	2.61E-04
227	Glycylleucine	peptides	5.11E-04
228	gamma-Glutamylserine	peptides	7.23E-04
229	Valylaspartic acid	peptides	9.74E-04
230	Aspartyl-Leucine	peptides	1.54E-03
231	Valylserine	peptides	2.49E-03
232	Carnosine	peptides	3.41E-03
233	Arginylasparagine	peptides	5.79E-03
234	L-prolyl-L-proline	peptides	6.01E-03
235	Phenylalanylproline	peptides	7.49E-03
236	gamma-Glutamylleucine	peptides	1.32E-02
237	Glutamylthreonine	peptides	1.42E-02
238	Hydroxyprolyl-Tyrosine	peptides	1.57E-02
239	Arginylhistidine	peptides	1.99E-02
240	Prolylhydroxyproline	peptides	2.13E-02
241	Prolyl-Threonine	peptides	2.51E-02
242	Gamma Glutamylglutamic acid	peptides	2.95E-02
243	Pyrocatechol	phenolic	1.13E-06
244	Acetaminophen	phenolic	8.26E-06
245	Isohomovanillic acid	phenolic	8.01E-05
246	Tyrosol	phenolic	9.72E-05
247	Homogentisic acid	phenolic	7.23E-04
248	Norepinephrine	phenolic	2.70E-03
249	4-Ethylphenol	phenolic	4.73E-02

250	6-Dimethylaminopurine	purine	2.26E-08
251	6-Methyladenine	purine	2.27E-04
252	Uric acid	purine	7.55E-04
253	1-Methylhypoxanthine	purine	3.15E-03
254	Xanthurenic acid	quinoline	2.15E-06
255	Kynurenic acid	quinoline	1.98E-04
256	Sphinganine 1-phosphate	sphingolipid	1.37E-02
257	Cholestenone	steroids	3.83E-09
258	3-Oxocholic acid	steroids	4.47E-08
259	Tetrahydrodeoxycorticosterone	steroids	9.27E-08
260	Androstanediol	steroids	4.20E-05
261	7-ketocholesterol	steroids	1.15E-02
262	Deoxycholic acid glycine conjugate	steroids	2.43E-02
263	Cholic acid	steroids	3.25E-02
264	17-Hydroxyprogesterone	steroids	4.44E-02
265	Beta-estradiol	steroids	4.58E-02
266	Lithocholic acid glycine conjugate	steroids	4.58E-02
267	D-Glucose 6-phosphate	sugars and their derivatives	6.96E-07
268	N-Acetyl-b-glucosaminylamine	sugars and their derivatives	2.41E-06
269	Deoxyribose 1-phosphate	sugars and their derivatives	4.76E-06
270	Fructose 1-phosphate	sugars and their derivatives	5.32E-06
271	D-Sedoheptulose 7-phosphate	sugars and their derivatives	1.94E-05
272	Methyl-beta-galactopyranoside	sugars and their derivatives	2.39E-05
273	D-Ribose 5-phosphate	sugars and their derivatives	2.66E-05
274	1,5-Anhydrosorbitol	sugars and their derivatives	1.54E-03
275	D-Mannitol 1-phosphate	sugars and their derivatives	4.29E-03
276	D-Glucarate	sugars and their derivatives	4.99E-03
277	D-Ribose	sugars and their derivatives	6.47E-03
278	Gluconic acid	sugars and their derivatives	6.96E-03
279	D-Glucose	sugars and their derivatives	1.11E-02
280	Maltol	sugars and their derivatives	1.19E-02
281	D-Glucosamine 6-phosphate	sugars and their derivatives	2.95E-02
282	Trehalose	sugars and their derivatives	3.05E-02
283	Glycyrrhetinic acid	terpenoids	1.72E-04
284	Ursolic acid	terpenoids	1.36E-03
285	Dihydroartemisinin	terpenoids	1.88E-03
286	Humulene	terpenoids	8.97E-03
287	Vitamin A	vitamins	1.19E-06
288	Pantothenic Acid	vitamins	3.80E-06
289	Pyridoxamine	vitamins	4.38E-04
290	Riboflavin	vitamins	6.63E-04
291	Nicotinic acid mononucleotide	vitamins	1.48E-03

292	Pyridoxal	vitamins	4.45E-03
293	L-Ascorbic acid	vitamins	1.03E-02
294	4-Pyridoxic acid	vitamins	1.57E-02

[1] Permutation based p value obtained from the JTK_circle algorithm.

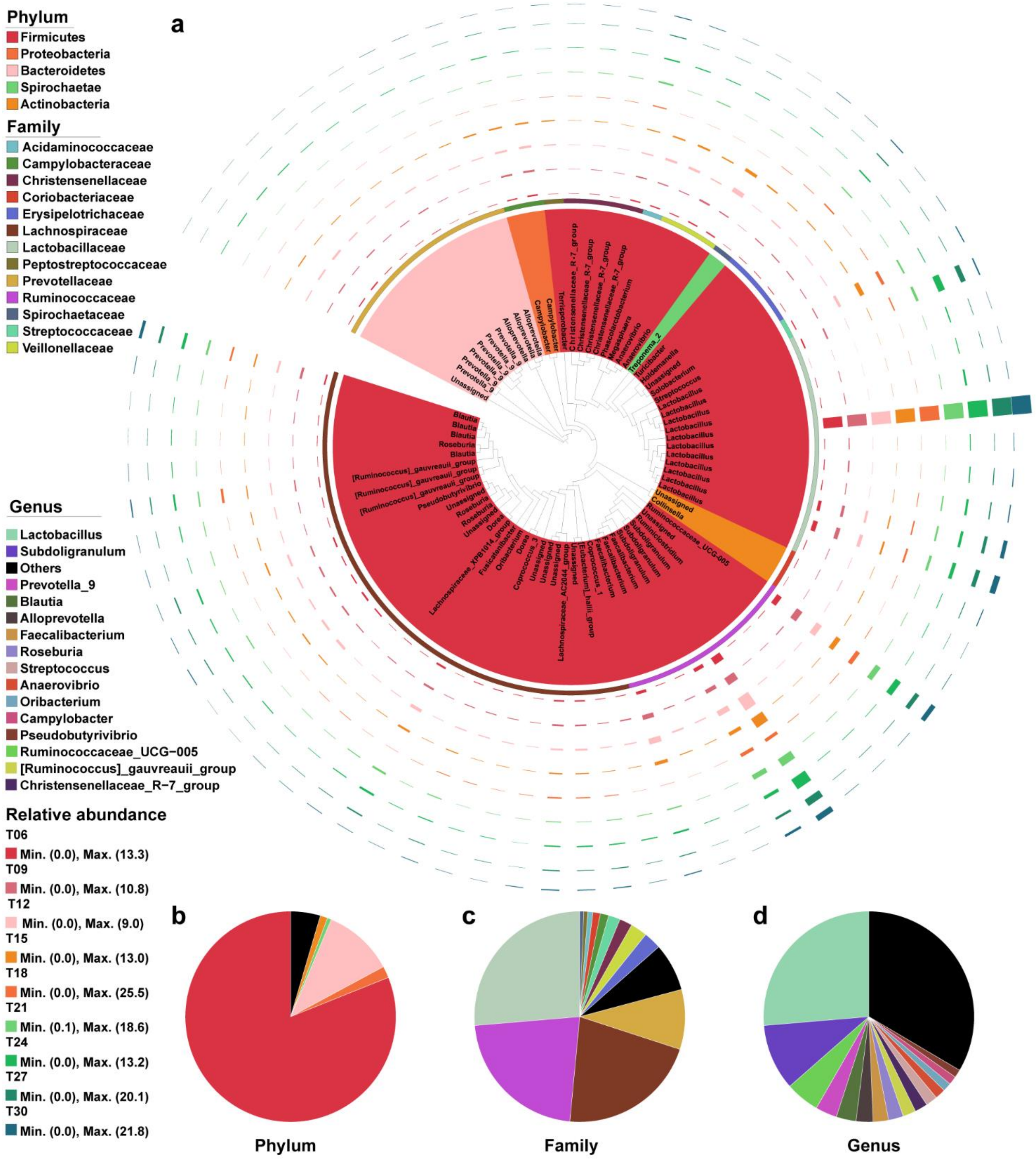

Supplementary Figure 1 Composition of the gut microbiota community in the colonic digesta of growing pigs

a Phylogenetic tree exhibiting the placement of key microbes based on 16S rRNA gene sequences. In the phylogenetic tree, each core ASV node was labeled with a genus name and was color-filled according to phylum. Each color in the inner-circle color strip represents a different microbial family.

Bar plots from inner to outer exhibit the mean relative abundance of each core ASV at T06, T09, T12, T15, T18, T21, T24, T27, and T30, respectively. ASV: amplicon single variant.

(b ~ d) Microbial composition at the phylum (B), family (C), and genus (D) level, respectively.

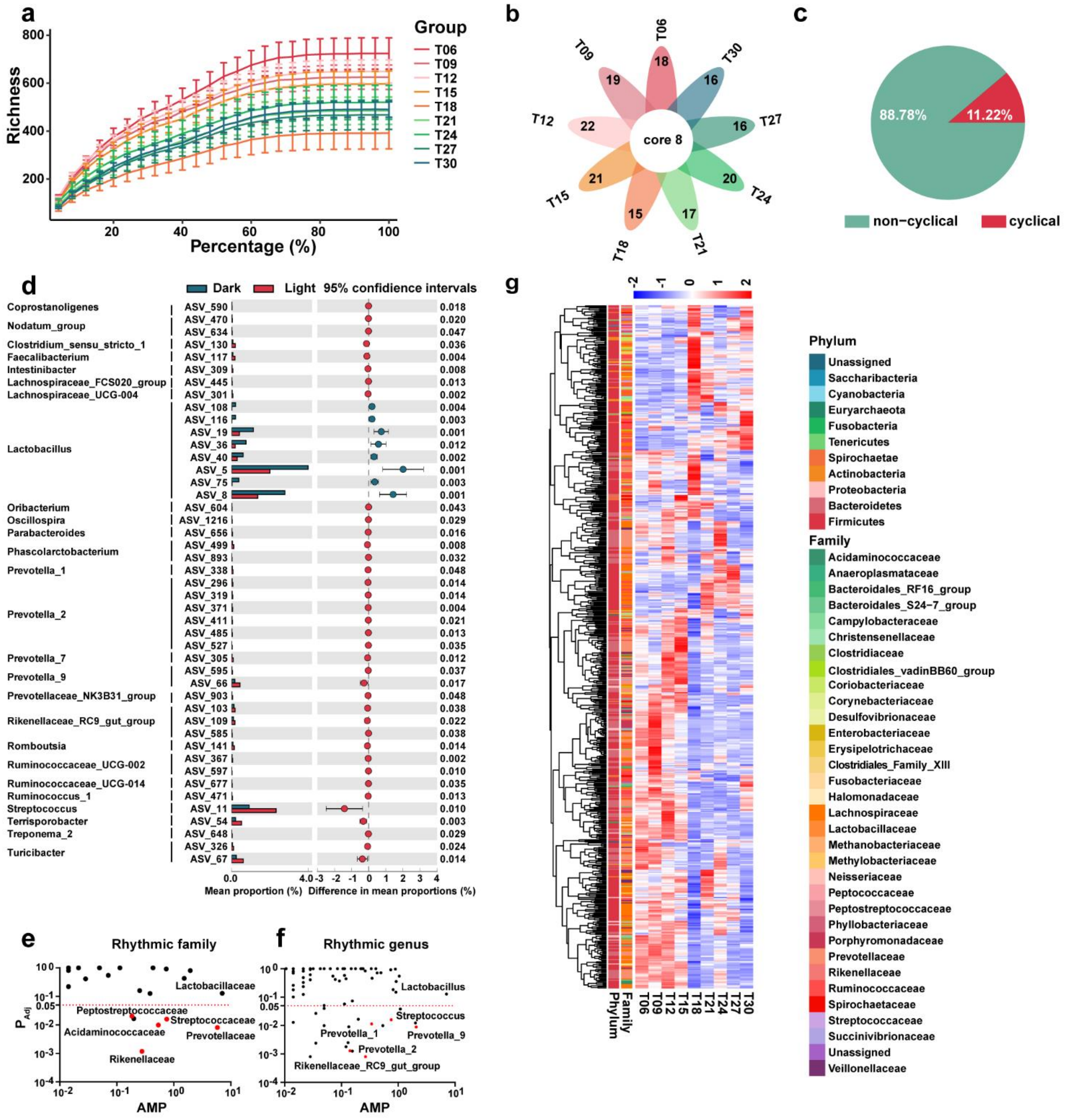

Supplementary Figure 2 Dynamic microbial profile in the colon ecology of growing pigs

a Rarefaction curve of each sampling timepoint

b Key microbial genera identified at different sampling timepoints.

c Pie chart showing the percentage of microbes with daily fluctuation at the ASV level.

d Diurnal difference of microbes between the light phase (T09, T12, T15 and T18) and the dark phase (T06, T21, T24, T27 and T30) at the ASV level.

e ~ f Cyclical microbes identified by JTK_circle analysis method at the family level (e) and the genus level (f).

g Heatmap depicting the relative abundance of each non-rhythmic ASV. The relative abundance of ASVs was normalized using Z-score methods. Row group annotation information from outside to inside represents its rhythmic fluctuation, corresponding phylum and family, respectively. ASV: amplicon single variant.

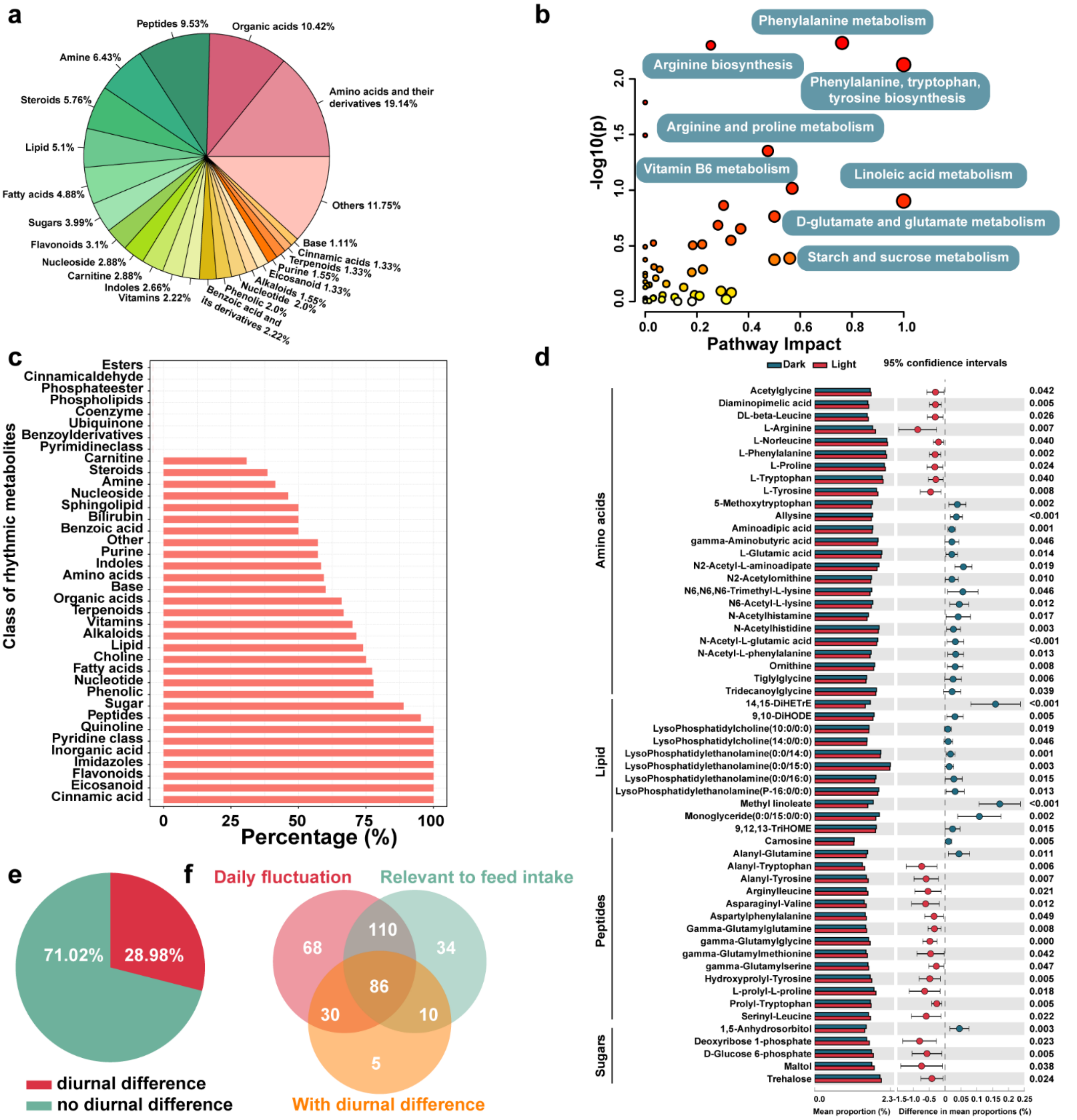

Supplementary Figure 3 Oscillation of colonic metabolites of growing pigs

a Pie chart depicting the composition of the metabolome profile.

b KEGG pathway enrichment analysis bubble chart of these cyclical metabolites.

c Percentage of cyclical metabolites of each class

d Diurnal difference of metabolites of different classes between the light phase (T09, T12, T15 and

T18) and the dark phase (T06, T21, T24, T27 and T30).

e Pie chart showing the percentage of metabolites with daily fluctuation.

f Venn diagram exhibiting the relationships of metabolites with rhythmicity, daily difference, and relevant with feed intake.

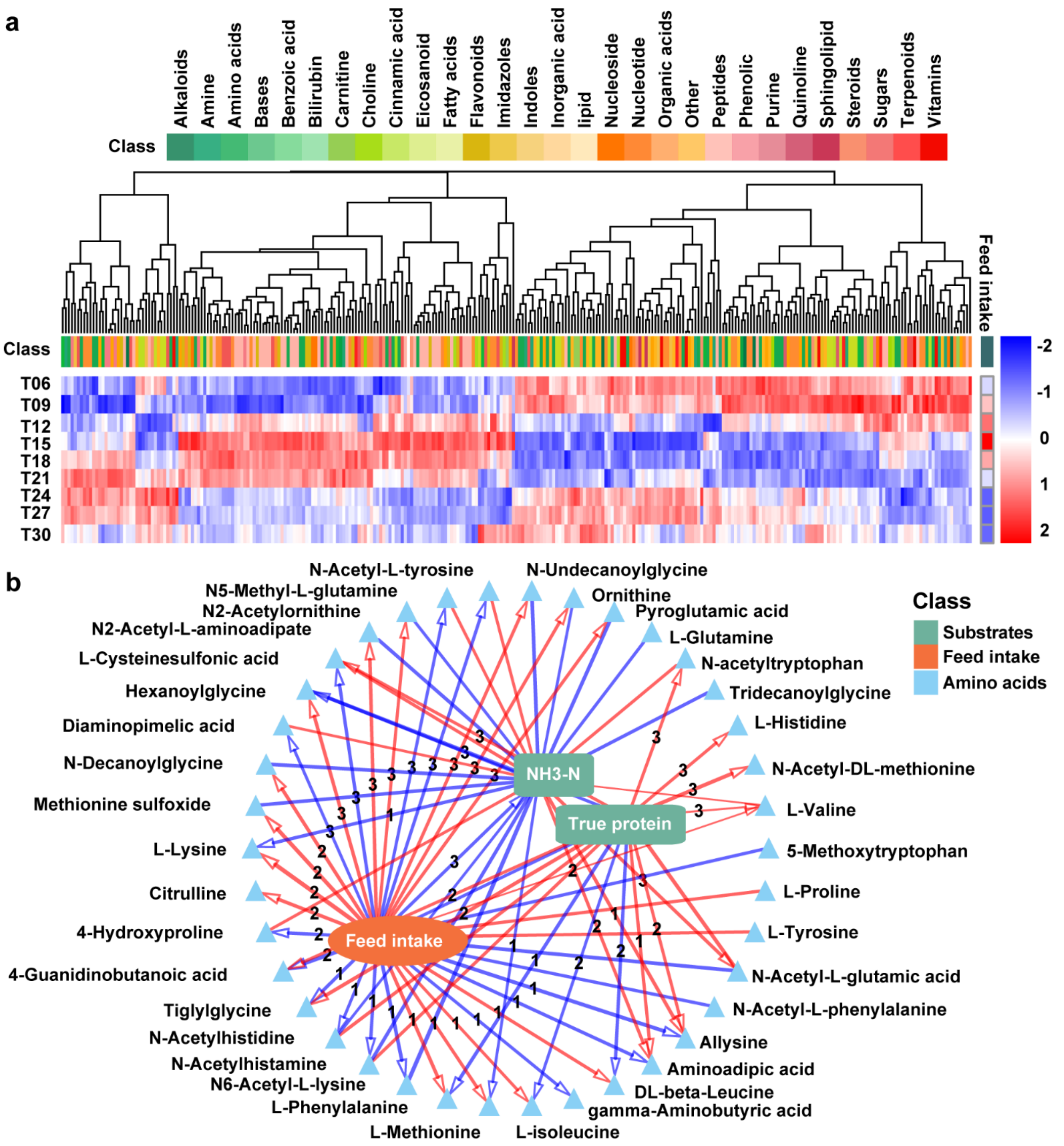

Supplementary Figure 4 Dynamic changes of cyclical metabolites and the correlations between amino acids, feed intake and the nutrient substrates

a Heatmap depicting the relative abundance of each cyclical metabolite. The relative abundance was normalized using Z-score methods. Row group annotation information represents the class.

b Dynamic correlation network between the colonic nitrogen substrates NH3N and true protein, the feed intake and the amino acid metabolites based on the extended local similarity analysis (eLSA)

method. Each edge represents a pairwise correlation with a local similarity (LS) > 0.5 and a $P < 0.05$. Edges in red color represent positive correlations whereas a blue edge represents a negative correlation. Edge label represents time shifts between colonic substrates and amino acid metabolites.

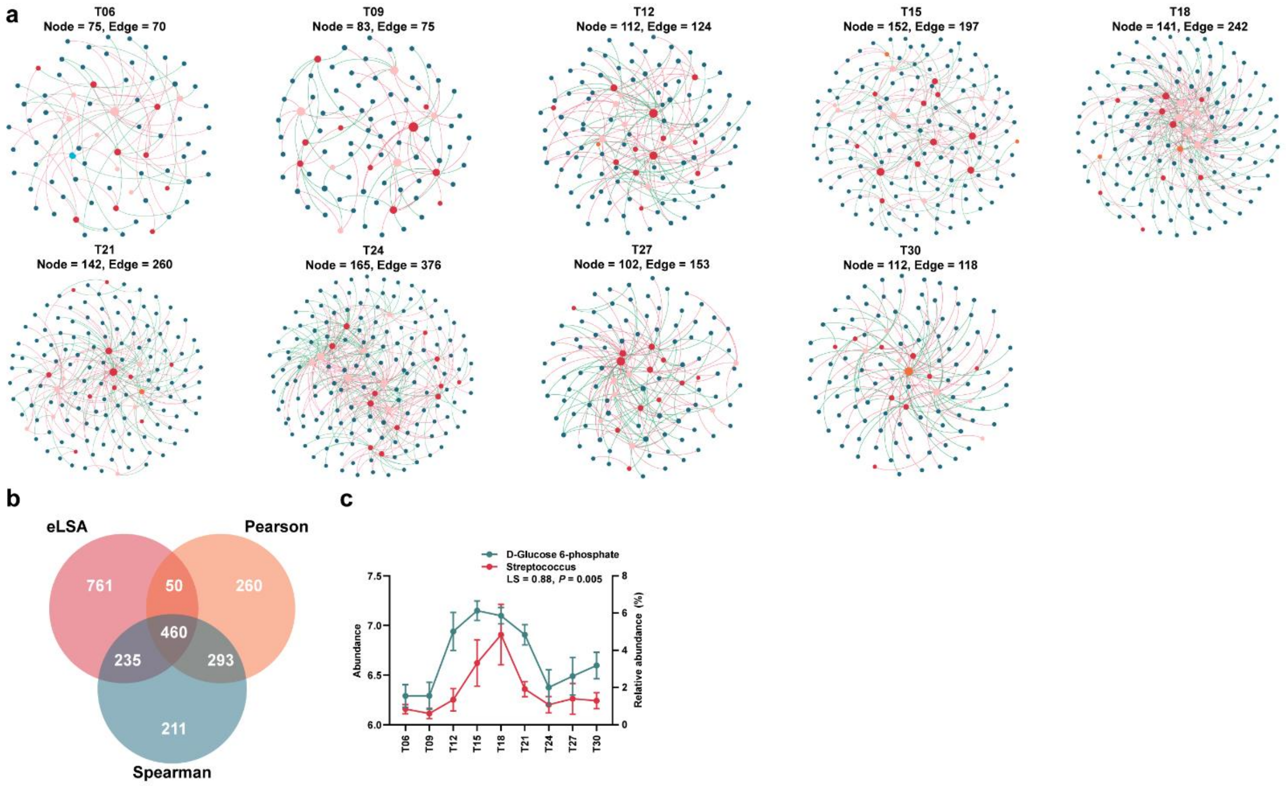

Supplementary Figure 5 The dynamic microbe-metabolite interaction in the colon of growing pigs within a day

a Microbe-metabolite interaction networks at different time points within a day. Each node in blue color represents one metabolite, whereas the other colored node exhibits a genus and is colored according to the phylum. Each edge represents a Spearman correlation with a r > 0.5 and a $P < 0.05$. Edges in red color represent positive correlations between a genus and a metabolite whereas a blue edge represents a negative correlation.

b Venn diagram exhibiting the number of pairwise correlations obtained from different computational methods of extended local similarity analysis (eLSA), Spearman, and Pearson.

c Dynamic changes of *Streptococcus* and metabolite D-Glucose 6-phosphate.

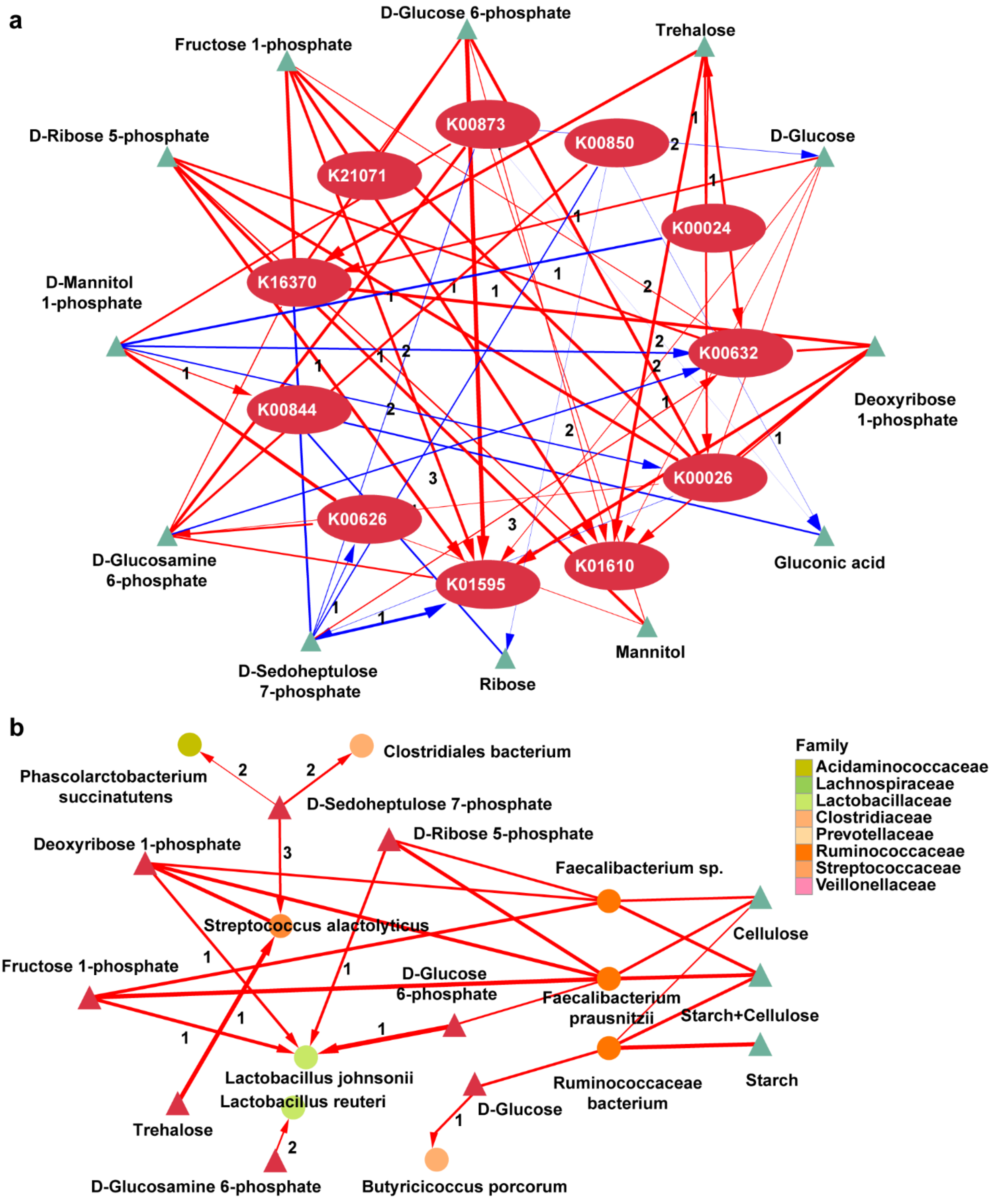

Supplementary Figure 6 Correlation analysis between the sugar metabolites, the KEGG genes and the microbes

a ~b Correlation between the sugar metabolites and the KEGG genes (a) and between contributory microbial species (b). Networks were constructed by eLSA method. Edges in red color represent

positive correlations whereas a blue edge represents a negative correlation. The width of the edge represents the values of the local similarity coefficients. The label of the edge represents time shift. Microbial taxa were colored according to the families.

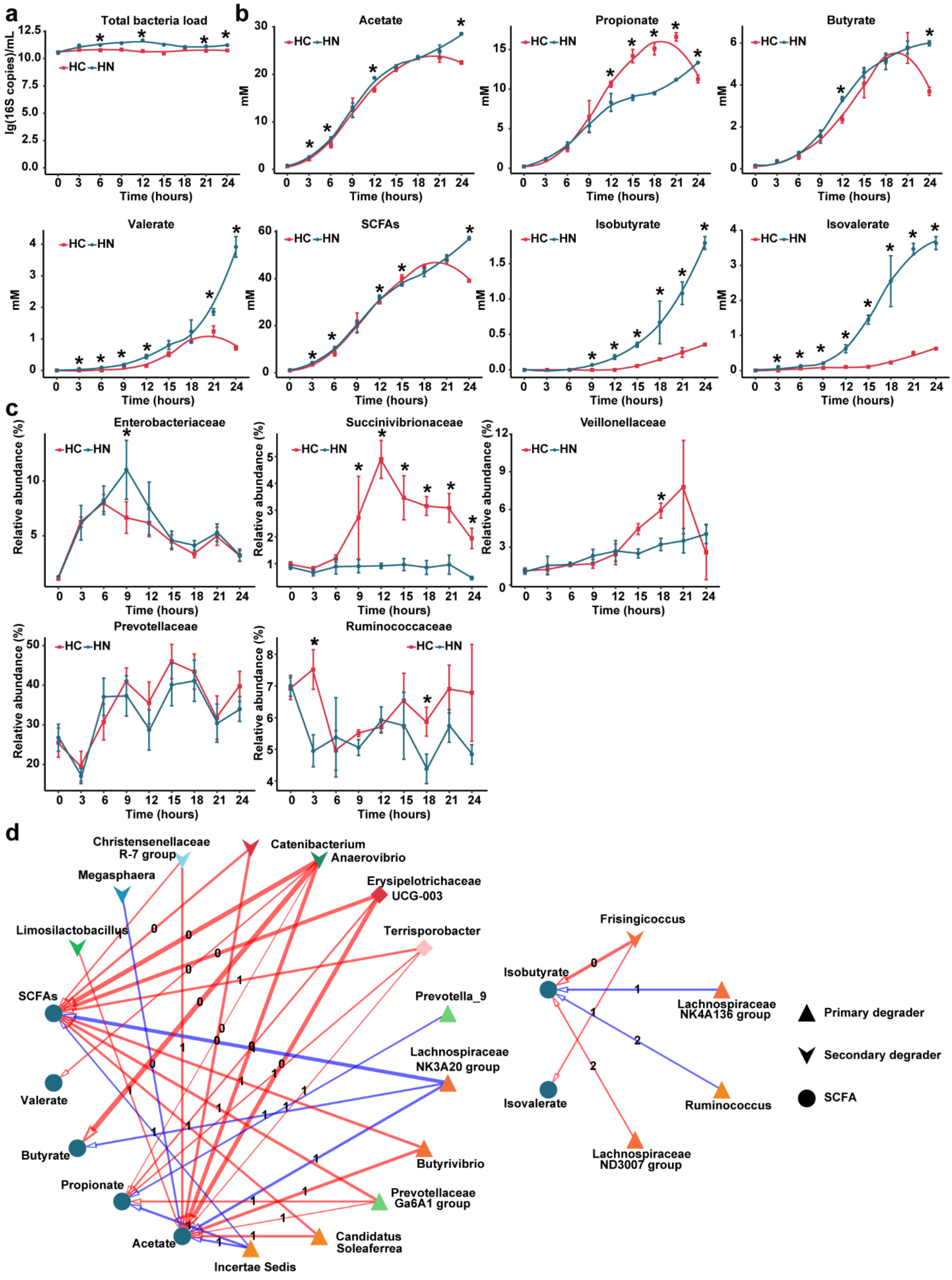

Supplementary Figure 7 Dynamic changes of microbes, and SCFAs and their correlations.

a Dynamic change of the total bacterial load at each sampling time point of the HC group and the HN group. * denotes a significant difference with a p-value lower than 0.05. HC = high carbohydrate group; HN = high nitrogen group. All data were presented as mean ± s.d.

b Dynamic fluctuations of SCFAs acetate, propionate, butyrate, isobutyrate, valerate, isovalerate and total SCFAs. The difference between different groups at each sampling timepoint was identified by a two-tailed t-test (n=4). * denotes a significant difference with a p-value lower than 0.05. HC = high carbohydrate group; HN = high nitrogen group. All data were presented as mean ± s.d.

c Dynamic fluctuations in relative abundance of Enterobacteriaceae, Succinivibrionaceae, Veillonellaceae, Prevotellaceae, and Ruminococcaceae. The difference between different groups at each sampling timepoint was identified by a two-tailed *t-test* (n=4). * denotes a significant difference with a p-value lower than 0.05. HC = high carbohydrate group; HN = high nitrogen group.

d Correlations between SCFAs and microbes belonging to different class. Networks were constructed by eLSA method. Each cell represents a pairwise correlation with a local similarity (LS) > 0.5 and a $P < 0.05$. The label on the cell represents time delay of a pairwise. Cells filled in red color represent positive correlations whereas a blue cell represents a negative correlation. The depth of the color represents the size of LS.
